# Near‐Infrared Conjugated Macrocyclic BODIPYs for Photothermal Cancer Therapy

**DOI:** 10.1002/anie.202511125

**Published:** 2025-09-27

**Authors:** Fei Cheng, Taotao Qiang, Mingli Li, Ruilong Li, Zhigao Wang, Tony D. James

**Affiliations:** ^1^ College of Bioresources and Materials Engineering Shaanxi Collaborative Innovation Center of Industrial Auxiliary Chemistry & Technology Shaanxi University of Science & Technology Xi'an 710021 China; ^2^ Department of Chemistry University of Bath Bath BA27AY United Kingdoms; ^3^ College of Pharmacy Xi'an Medical University Xi'an 710021 China; ^4^ School of Chemistry and Chemical Engineering Henan Normal University Xinxiang 453007 China

**Keywords:** Non‐radiative transition, Photothermal conversion efficiency, Photothermal therapy, Radiative transition

## Abstract

With this research we developed a cyclization strategy that effectively converts radiative transitions (RTs) to non‐radiative transitions (NRTs) during the decay process of BODIPY‐based photosensitizers. Compared to BODIPY monomers, cyclization leads to a significant decrease in the HOMO–LUMO energy gap and a significant increase in the HOMO energy. In particular, the absorption spectrum of **7a** exhibits a significant redshift, with the maximum absorption wavelength reaching 794 nm. Photophysical characterization indicates that the macrocyclic BODIPY derivatives **7(a–d)** exhibit a reduced RT process. While, the crystal structure and theoretical calculations suggest that the molecular ring distortion enhances the intersystem crossing (ISC) ability of the macrocyclic BODIPY derivatives **7(a–d)**. In addition **7a‐NPs** constructed using DSPE‐mPEG_2000_ encapsulation exhibit excellent water solubility, stability, and photothermal conversion efficiency (44.6%). The photothermal therapeutic performance of **7a‐NPs** was evaluated through in vitro cell and in vivo mice experiments. The results indicated that **7a‐NPs** could be enriched at the tumor site and exhibited strong tumor ablation ability using near‐infrared radiation. Our findings suggest that the regulation of the RT to NRT conversion using an alkali‐induced cyclization reaction is a useful strategy for preparing efficient photo‐thermal conversion materials based on BODIPY.

## Graphical abstract

Nucleophilic substitution‐cyclization‐dehydrogenation reactions generate a photosensitizer exhibiting enhanced non‐radiative transitions, resulting in excellent photothermal therapeutic effects.

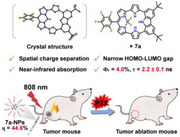

## Introduction

Cancer is one of the major diseases threatening human health and quality of life because of its high incidence, difficulty to cure, and high mortality.^[^ ^1^, ^2^, ^3^, ^4^, ^5^, ^6^, ^7^, ^8^ ^]^ Traditional treatment methods such as surgery, chemotherapy, and radiotherapy, all exhibit significant drawbacks such as severe trauma, notable side effects, and high recurrence rates, and as such are gradually being replaced by less invasive and precise techniques like photodynamic therapy (PDT) and photothermal therapy (PTT) that offer simple operation and accurate targeting.^[^ ^9^, ^10^, ^11^, ^12^, ^13^, ^14^, ^15^ ^]^ PDT largely depend on photosensitizers converting the first excited singlet (S_1_) to the triplet state (T_n_) through intersystem crossing (ISC), and then through energy transfer of the first excited triplet (T_1_) to produce reactive oxygen species that then kills tumor cells.^[^ ^16^, ^17^, ^18^, ^19^ ^]^ On the other hand, PTT primarily relies on the release of heat energy to kill tumor cells generated from the photosensitizers as they return from the S_1_ state to the ground state (S_0_).^[^ ^20^, ^21^, ^22^, ^23^ ^]^

Despite the significant achievements of PDT for tumor treatment, the hypoxic conditions commonly found in tumor microenvironments (PDT primarily relies on molecular oxygen) can reduce the efficacy of PDT.^[^ ^24^, ^25^ ^]^ On the other hand, PTT is less influenced by the tumor microenvironment during the tumor treatment process and can be monitored in real‐time using thermal imaging systems, which exhibit high temperature sensitivity and controllability.^[^ ^26^, ^27^ ^]^ Therefore, PTT holds greater potential for applications in the field of tumor treatment.^[^ ^28^, ^29^, ^30^, ^31^, ^32^, ^33^, ^34^, ^35^, ^36^ ^]^ PTT typically utilizes near‐infrared light excitation, which has strong penetration ability and is harmless to normal tissues.^[^ ^37^, ^38^, ^39^, ^40^ ^]^ Therefore, the key to efficient PTT lies in the development of photosensitizers with near‐infrared absorption and high photothermal conversion efficiency (PCE). BODIPY is a class of conjugated organic photosensitizers that contains two N→B bonds. Its unique photophysical properties enable its pivotal role in cancer treatment. However, the shorter absorption wavelength and lower PCE of BODIPY limit its application for tumor PTT.^[^ ^28^ ^]^ In the development of long‐wavelength light absorbing photosensitizers, researchers have expanded the molecular π‐conjugated system to enable BODIPY‐like photosensitizers to exhibit long‐wavelength absorption, primarily involving the construction of aromatic conjugated systems and chain conjugated systems.^[^ ^41^, ^42^ ^]^ Building on this foundation, various near‐infrared BODIPY‐based photosensitizers with high NRT capabilities have been developed through strategies such as introducing CF_3_ functional groups at the *meso* position of the BODIPY core, constructing conjugated polymers, and adjusting the aggregation of nanomaterials. These photosensitizers have been successfully applied in the field of PTT, achieving effective tumor eradication.^[^ ^43^, ^44^, ^45^, ^46^, ^47^, ^48^ ^]^ However, these compounds still face challenges, such as complex synthesis and difficulties in structural characterization. Additionally, the targeting performance of BODIPY nanomaterials is relatively poor, resulting in suboptimal drug distribution during phototherapy and inevitable side effects.^[^ ^28^ ^]^ Therefore, developing near‐infrared BODIPY‐based photosensitizers that are easy to synthesize, have well‐defined structures, high NRT capabilities, and improved targeting properties is crucial for achieving efficient PTT.

**Figure 1:**
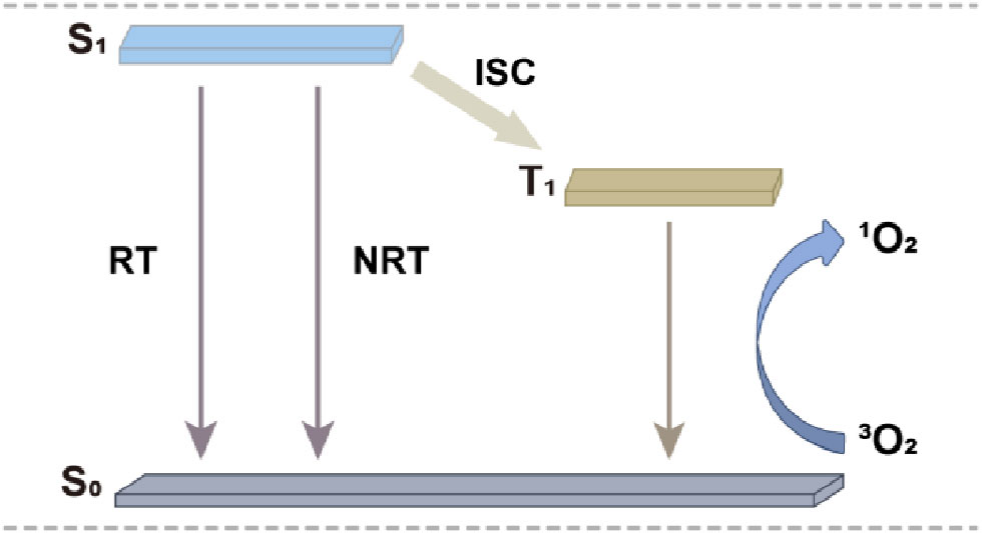
Schematic diagram illustrating the decay pathways of a photosensitizer's excited state returning to the ground state.

Indeed, the excited state of photosensitizers undergoes relaxation back to the S_0_ via three main pathways: RT, NRT, and ISC (Figure 1). To enhance the NRT capability of BODIPY‐based photosensitizers and improve their photothermal treatment performance, it is necessary to inhibit their RT process. Currently, there are few reports on the application of macrocyclic BODIPY‐based photosensitizers with near‐infrared absorption, low RT, and weak ISC capabilities in the field of PTT. Therefore, it is of great significance to develop macrocyclic BODIPY‐based photosensitizers with strong NRT abilities and near‐infrared absorption, and to explore their practical application in PTT (Table S5 and Figure S74).

Herein, we synthesized near‐infrared BODIPY derivatives **7(a–d)** containing a macrocyclic π‐conjugated system, where the generality of the cyclization strategy has been verified using different BODIPY monomers. Theoretical calculations and experimental trials were used to verify whether the electron‐absorbing properties of the BODIPY unit can promote intramolecular charge separation, reduce the HOMO–LUMO gap, and expand the light absorption range. In addition, the introduction of a C_6_F_5_ group (which enhances intramolecular charge separation by its electron‐withdrawing nature) further reduces the HOMO–LUMO gap and increases the molecular absorption range. As the absorption spectra undergoes a red‐shift, the intrinsic fluorescence of the molecule decreases significantly, and the fluorescence quantum yield of compounds **7(a–d)** decreases sharply (Table S4 and Figure S55). By combining theory with practice, the origin and strength of intersystem crossover ability in compounds **7(a–d)** were elucidated. Nanoparticles assembled from **7a** and 1,2‐distearoyl‐sn‐glycero‐3‐phosphoethanolamine‐*N*‐[methoxy‐(polyethyleneglycol)‐2000] (DSPE‐mPEG_2000_) demonstrate excellent PCE (44.6%), stability, PTT performance and biocompatibility in the near‐infrared window.^[^ ^49^ ^]^ Therefore, this study provides a useful strategy for developing near‐infrared BODIPY‐based photosensitizers exhibiting low RT and ISC with high NRT capability.

## Results and Discussion

### Molecular Structures

Long‐wavelength light absorbing photosensitizers containing cyclic π‐conjugated systems exhibit higher stability. Furthermore, the spontaneous fluorescence of these photosensitizers tends to decrease as the absorption wavelength of the photosensitizer redshifts.^[^ ^50^ ^]^ Inspired by this, we began to explore photosensitizers based on cyclic π‐conjugated structures that absorb in the long wavelength range.

**Figure 2:**
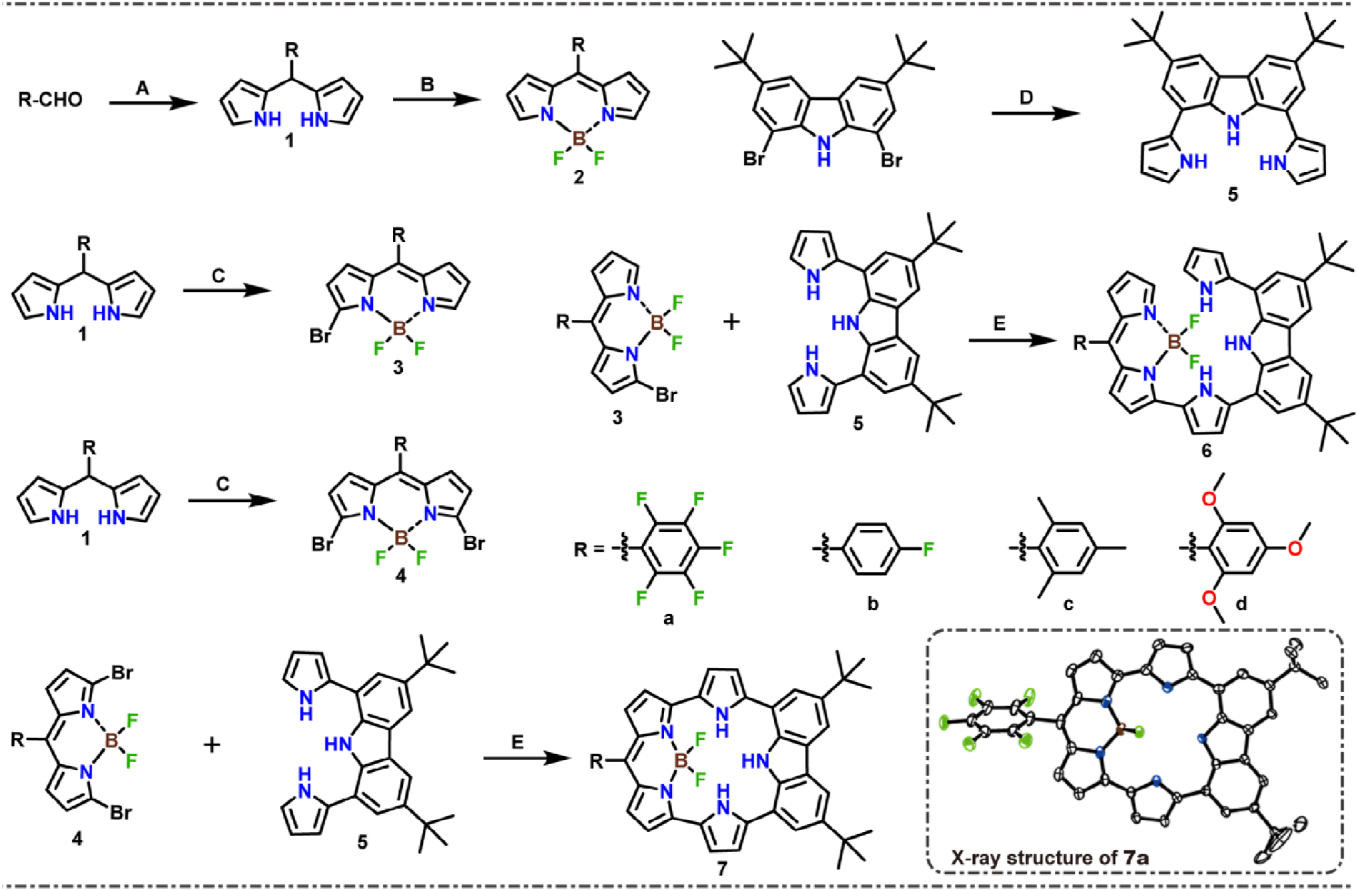
Schematic diagram of the synthesis of BODIPY derivatives **6(a–d)** and **7(a–d)** (**A**: Pyrrole, trifluoroacetic acid, N_2_, room temperature, 5–60 min; **B**: 2, 3‐dichloro‐5, 6‐dicyano‐1, 4‐benzoquinone, dichloromethane, room temperature, 1 h; Triethylamine, boron trifluoride ether, 1, 2‐dichloroethane, N_2_, 85 °C, 4 h; **C**: *N*‐bromosuccinimide, 2, 3‐dichloro‐5, 6‐dicyano‐1, 4‐benzoquinone, tetrahydrofuran, N_2_, ‐78 °C, 2 h; Triethylamine, boron trifluoride ether, 1, 2‐dichloroethane, N_2_, 85 °C, 4 h; **D**: pyrrole‐2‐boronic acid pinacol ester, tri(dibenzalacetone)dipalladium, triphenylphosphine, cesium carbonate, cesium fluoride, toluene, *N, N*‐dimethylformamide, N_2_, 115 °C, 48 h; **E**: Cesium carbonate, *p*‐xylene, N_2_, 135 °C, 24–48 h), as well as the X‐ray structure of **7a** (50% thermal ellipsoids; H atoms omitted).

In 2024, Song's group synthesized compound **7c**, which contains both BODIPY structural units and carbazole units, and conducted a detailed evaluation of the molecular structure (Figure 2).^[^ ^51^ ^]^ Additionally, preliminary studies were performed on the photophysical properties of **7c** (without investigating the excited‐state properties of the molecule and its practical applications). Building on this, we chose to introduce substituents with different electron‐donating/withdrawing properties at the meso position of the BODIPY core to modulate the photophysical properties of the molecule. Moreover, since both the heteroatoms in the BODIPY and carbazole structures are nitrogens, we selected pyrrole as the bridging unit between the BODIPY and carbazole units in the macrocyclic structure (to maintain a high degree of atomic similarity enabling us to more easily uncover the rules governing the modulation of the molecule's photophysical properties). In our research, BODIPY monomers **2** and non‐cyclic BODIPY derivatives **6** were used as control compounds.

### Synthesis and Characterization

The molecular structure of macrocyclic BODIPY derivatives **7(a–d)** is depicted in Figure 2. For more detailed synthetic information, please refer to the Supporting Information. First, meso‐aryldipyrroles **1(a–d)** were synthesized via acid‐catalyzed condensation using aromatic aldehyde and pyrrole as substrates, with yields of 48%–58%. Then, BODIPY monomers **2(a–d)** were synthesized from meso‐aryldipyrroles **1(a–d)** with yields of 64%–68% through 2,3‐dichloro‐5,6‐dicyanobenzoquinone‐induced intramolecular double bond oxidation rearrangement and boron trifluoride diethyl etherate‐assisted pyrrole nitrogen fluoroboration. Simultaneously, starting from meso‐aryldipyrroles **1(a–d)** as the substrate, a series of reactions including *N*‐bromosuccinimide‐mediated pyrrole α‐bromination, 2,3‐dichloro‐5,6‐dicyanobenzoquinone‐induced intramolecular double bond oxidation rearrangement, and boron trifluoride diethyl etherate‐assisted pyrrole nitrogen borylation were carried out to synthesize α‐bromo BODIPYs **3(a–d)** with yields of 35%–42% and **4(a–d)** with yields of 45%–51%. Next, 1,8‐dibromo‐3,6‐di‐tert‐butyl‐9H‐carbazole and pyrrole‐2‐boronic acid pinacol ester were used as starting materials, and a palladium‐catalyzed Suzuki‐Miyaura cross‐coupling reaction was employed to synthesize 1,8‐dipyrryl‐3,6‐di‐tert‐butyl‐9H‐carbazole **5** with a yield of 35.4%. Finally, large π‐conjugated macrocyclic BODIPY derivatives **7(a–d)** were synthesized with yields of 27%–32% using compounds **4(a–d)** and **5** as substrates through nucleophilic substitution‐cyclization‐dehydrogenation reactions.^[^ ^51^ ^]^ At the same time, using **3(a–d)** and **5** as reaction substrates, non‐cyclic BODIPY derivatives **6(a–d)** were synthesized under similar reaction conditions, with yields of 8%–13%. Compared to the BODIPY monomers **2(a–d)** and non‐cyclic BODIPY derivatives **6(a–d)**, the macrocyclic BODIPY derivatives **7(a–d)** exhibit excellent stability in both air and solution, as well as high thermal stability (decomposition temperature above 400 °C, Figure S1). Additionally, the structures of compounds **6(a–d)** and **7(a–d)** were confirmed using NMR and HR‐MS techniques (Figures S19–S46).

Fortunately, a single crystal of **7a** was obtained using liquid‐phase diffusion (CHCl_3_/MeCN). X‐ray crystallography revealed that **7a** possesses a distorted cyclic conjugated structure, with a large dihedral angle of 74.54(3)° between the C_6_F_5_ substituent and the BODIPY core unit (ABC plane) due to steric hindrance. In addition, the molecular structure of the BODIPY core unit (ABC plane) is distorted due to the torsional strain and steric effects, resulting in dihedral angles of 17.08(4)°, 28.48(15)°, and 13.35(12)° between the ABC plane and the left pyrrole unit (D plane), right pyrrole unit (E plane), and carbazole unit (FGH plane), respectively. (Figures 2 and S47). We optimized the geometric structures of both the BODIPY monomers, non‐cyclic BODIPY derivatives and macrocyclic BODIPY derivatives at the PBE0/6‐31G (d, p) level to further investigate the structural characteristics of macrocyclic BODIPY derivatives. The results of the geometry optimization indicate that the unmodified BODIPY core unit (ABC plane) exhibits good planarity, while the core units of the non‐cyclic BODIPY derivatives and macrocyclic BODIPY derivatives also lie in the same plane, suggesting that they are not affected by these modifications (Figures S48–S50). The optimized geometric structure of the macrocyclic BODIPY derivatives exhibited similar twisted features to the crystal structure of **7a** (Figure S50 and Table S2).

**Figure 3:**
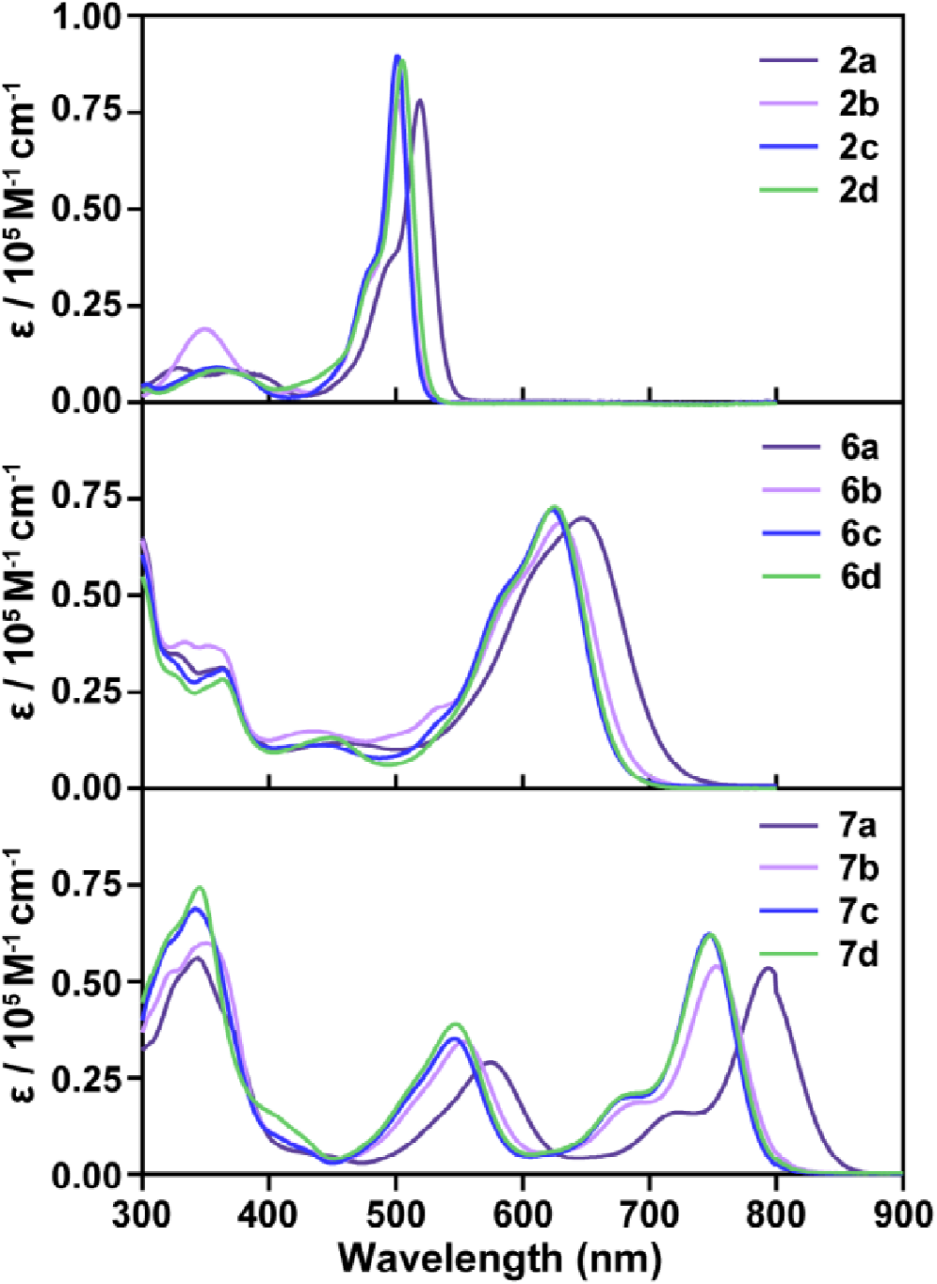
UV–vis‐NIR absorption spectra of BODIPY monomers (**2a**, **2b**, **2c,** and **2d**), non‐cyclic BODIPY derivatives (**6a**, **6b**, **6c,** and **6d**) and macrocyclic BODIPY derivatives (**7a**, **7b**, **7c,** and **7d**) in dichloromethane.

The absorption spectra of photosensitizers exhibit a redshift with increasing conjugation of the molecular system. As shown in Figure 3, the maximum absorption wavelength of BODIPY monomers in the absorption spectra is located in the visible region (**2a** at 519 nm, **2b** at 501 nm, **2c** at 501 nm, and **2d** at 505 nm). Due to the formation of a macrocyclic π‐conjugated system within the molecule, **7(a–d)** exhibit significant redshifts of absorption spectra, extending into the near‐infrared region (**7a** at 794 nm, **7b** at 752 nm, **7c** at 747 nm, and **7d** at 748 nm). Compared to the BODIPY monomers **2(a–d)** and the macrocyclic BODIPY derivatives **7(a–d)**, the absorption spectra of the non‐cyclic BODIPY derivatives **6(a–d)** exhibit a certain degree of red shift. However, their maximum absorption peaks still fall within the visible region (**6a** at 647 nm, **6b** at 629 nm, **6c** at 623 nm, and **6d** at 625 nm). This result indicates that the formation of an intramolecular cyclic π‐conjugated system is favorable for a red shift of the absorption spectra. According to the absorption spectra, the BODIPY monomers **2(a–d)** exhibit a relatively large optical band gap (*E*_g_^opt^ = 2.39–2.48 eV). In comparison, the optical band gap of the non‐cyclic BODIPY derivatives **6(a–d)** is decreased (*E*_g_^opt^ = 1.92–1.99 eV). The formation of an intramolecular cyclic conjugated system further reduces the optical band gap of the compounds **7(a–d)** (*E*_g_^opt^ = 1.56–1.66 eV). As shown in Figure S53, TD‐DFT calculations were performed on the geometrically optimized structure at the PBE0/6‐31G (d, p) level, confirming the near‐infrared absorption of macrocyclic BODIPY derivatives **7(a–d)** and its relatively small HOMO–LUMO energy gap (*E*_g_^DFT^ = 1.92–2.07 eV). Due to the high structural similarity between the non‐cyclic BODIPY derivatives **6(a–d)** and the macrocyclic BODIPY derivatives **7(a–d)**, their HOMO–LUMO band gap (*E*_g_^DFT^ = 1.87–2.14 eV) differences are close to those of the macrocyclic BODIPY derivatives **7(a–d)** (Figure S52). In general, the trend of the band gap obtained from theoretical calculations, as well as the optical band gap variations inferred from absorption spectra, are consistent. The HOMO of the non‐cyclic BODIPY derivatives **6(a–d)** is located within the entire π‐conjugated system of the molecule, while the LUMO is primarily concentrated on the BODIPY structural unit and the bridging pyrrole unit. Similarly, the HOMO of the macrocyclic BODIPY derivatives **7(a–d)** is located within the entire macrocyclic π‐conjugated system, while the LUMO is primarily concentrated on the BODIPY structural unit and the two bridging pyrrole units. The spatial separation of the HOMO–LUMO in compounds **6(a–d)** and **7(a–d)** indicates the presence of a D (electron donor: carbazole unit)/A (electron acceptor: BODIPY structural unit) features within compounds **6(a–d)** and **7(a–d)**. Subsequently, the absorption spectra of compounds **6(a–d)** and **7(a–d)** in different solvents were evaluated, revealing that the maximum absorption wavelength of compounds **6(a–d)** and **7(a–d)** blue‐shifts as solvent polarity increases (Table S3 and Figure S54).^[^ ^52^ ^]^ In addition the emission spectra of BODIPY monomers, non‐cyclic BODIPY derivatives and macrocyclic BODIPY derivatives were determined at room temperature. As shown in Table S4 and Figure S55, non‐cyclic BODIPY derivatives **6(a–d)** exhibit a lower fluorescence quantum yield compared to BODIPY monomers **2(a–d)**. Compared to the non‐cyclic BODIPY derivatives **6(a–d)**, the macrocyclic BODIPY derivatives **7(a–d)** exhibit an improvement in fluorescence quantum yield, although still remaining at a relatively low level. The lower fluorescence quantum yield indicates that non‐cyclic BODIPY derivatives **6(a–d)** and macrocyclic BODIPY derivatives **7(a–d)** exhibit weak RT capability.

### ISC Capability and Mechanism

**Figure 4:**
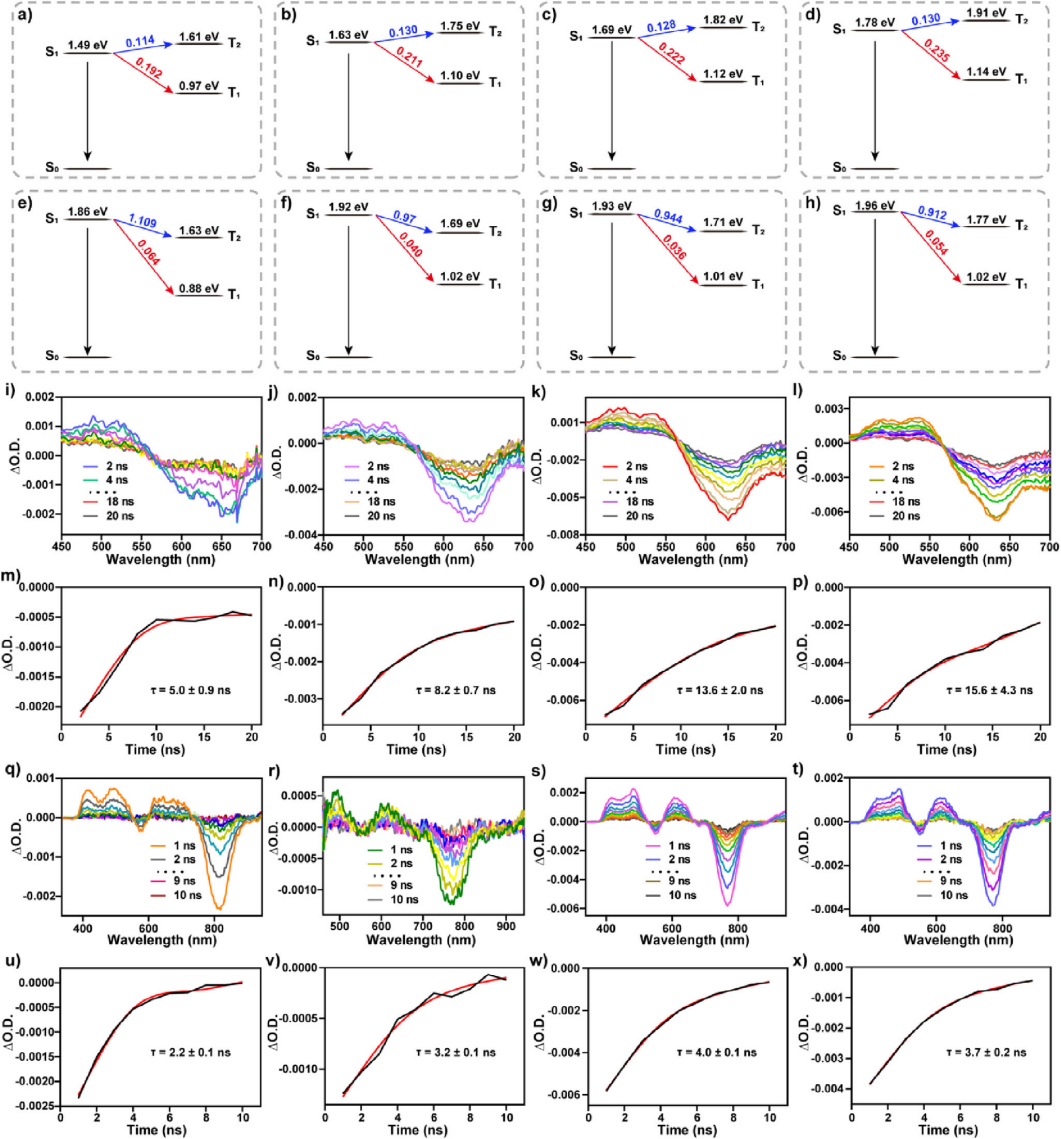
a)–d) SOC constants of non‐cyclic BODIPY derivatives **6(a–d)** between single and triplet energy levels (unit: cm^−1^); e)–h) SOC constants of macrocyclic BODIPY derivatives **7(a–d)** between single and triplet energy levels (unit: cm^−1^); i)–l) Nanosecond transient absorption spectra of non‐cyclic BODIPY derivatives **6(a–d)** (solvent is dichloromethane, *λ*_ex_ = 400 nm, *c* = 10^−4^ M); m)–p) Lifetime decay curve of non‐cyclic BODIPY derivatives **6(a–d)**; q)–t) Nanosecond transient absorption spectra of macrocyclic BODIPY derivatives **7(a–d)** (solvent is dichloromethane, *λ*_ex_ = 556 nm, *c* = 10^−4^ M); u)–x) Lifetime decay curve of macrocyclic BODIPY derivatives **7(a–d)**.

In summary, the excited state of non‐cyclic BODIPY derivatives **6(a–d)** and macrocyclic BODIPY derivatives **7(a–d)** mainly decays through the ISC and NRT pathways. To verify the possibility of ISC, TD‐DFT calculations were performed using the ORCA program to calculate the spin‐orbit coupling matrix elements. Based on previous studies, it is known that unmodified BODIPY monomers do not possess ISC capability (SOC value is zero).^[^ ^53^ ^]^ Subsequently, the SOC constants for the S_1_→T_1_ and S_1_→T_2_ transitions in the non‐cyclic BODIPY derivatives **6(a–d)** and the macrocyclic BODIPY derivatives **7(a–d)** were calculated using the spin‐orbit coupling matrix elements, with results showing positive values. This indicates that both compounds **6(a–d)** and **7(a–d)** possess ISC capability and the characteristics of triplet states. Compared to the non‐cyclic BODIPY derivatives **6(a–d)**, the macrocyclic BODIPY derivatives **7(a–d)** exhibit lower SOC constants (Figure 4a–h). This result indicates that the macrocyclic BODIPY derivatives **7(a–d)** have reduced ISC capability and triplet state characteristics. To further quantify the ISC capability and triplet state properties of compounds **6(a–d)** and **7(a–d)**, the triplet lifetimes of compounds **6(a–d)** and **7(a–d)** can be determined using nanosecond transient absorption spectroscopy (Figure 4i–l,q–t). The triplet lifetimes of compounds **6(a–d)** and **7(a–d)** were determined by monitoring the change in absorbance intensity at the maximum absorption wavelength over time (**6a**: 5.0 ± 0.9 ns; **6b**: 8.2 ± 0.7 ns; **6c**: 13.6 ± 2.0 ns; **6d**: 15.6 ± 4.3 ns; **7a**: 2.2 ± 0.1 ns; **7b**: 3.2 ± 0.1 ns; **7c**: 4.0 ± 0.1 ns; **7d**: 3.7 ± 0.2 ns) (Figure 4m–p,u–x). The quantum yield of singlet oxygen (^1^O_2_), as a representative species for triplet energy conversion, to some extent represents the triplet state performance. Therefore, the quantum yields of ^1^O_2_ in compounds **6(a–d)** and **7(a–d)** were determined using 9,10‐anthracenediylbis(methylene)dimalonic acid (ABDA) as a scavenger for ^1^O_2_. In the presence of compounds **6(a–d)** and **7(a–d)**, the optical absorption intensity of ABDA in DMSO decreases with increasing irradiation time. This indicates that compounds **6(a–d)** and **7(a–d)** can react with ^3^O_2_ to generate ^1^O_2_, which then reacts with ABDA, leading to a decrease in its optical absorption intensity. Simultaneously, using methylene blue (MB) as a reference with a ^1^O_2_ quantum yield of 52%, the ^1^O_2_ quantum yields of compounds **6(a–d)** and **7(a–d)** were calculated to be 11.1%, 11.3%, 16.8%, 18.2%, 10.9%, 7.1%, 15.3%, and 16% respectively (Figures S56–S58).^[^ ^54^ ^]^ Based on theoretical calculations, nanosecond transient absorption tests, and ^1^O_2_ quantum yield measurements, it can be concluded that compounds **7(a–d)** possess weak ISC capability and triplet state properties. This result suggests that the formation of an intrinsic cyclic π‐conjugated system within compounds **7(a–d)** further diminishes their ISC capability and triplet state characteristics. In summary, only a very small portion of the excited states of compounds **7(a–d)** will decay through the RT and ISC pathways.

### Photothermal Conversion Performance and in Vitro Cell Experiments

**Figure 5:**
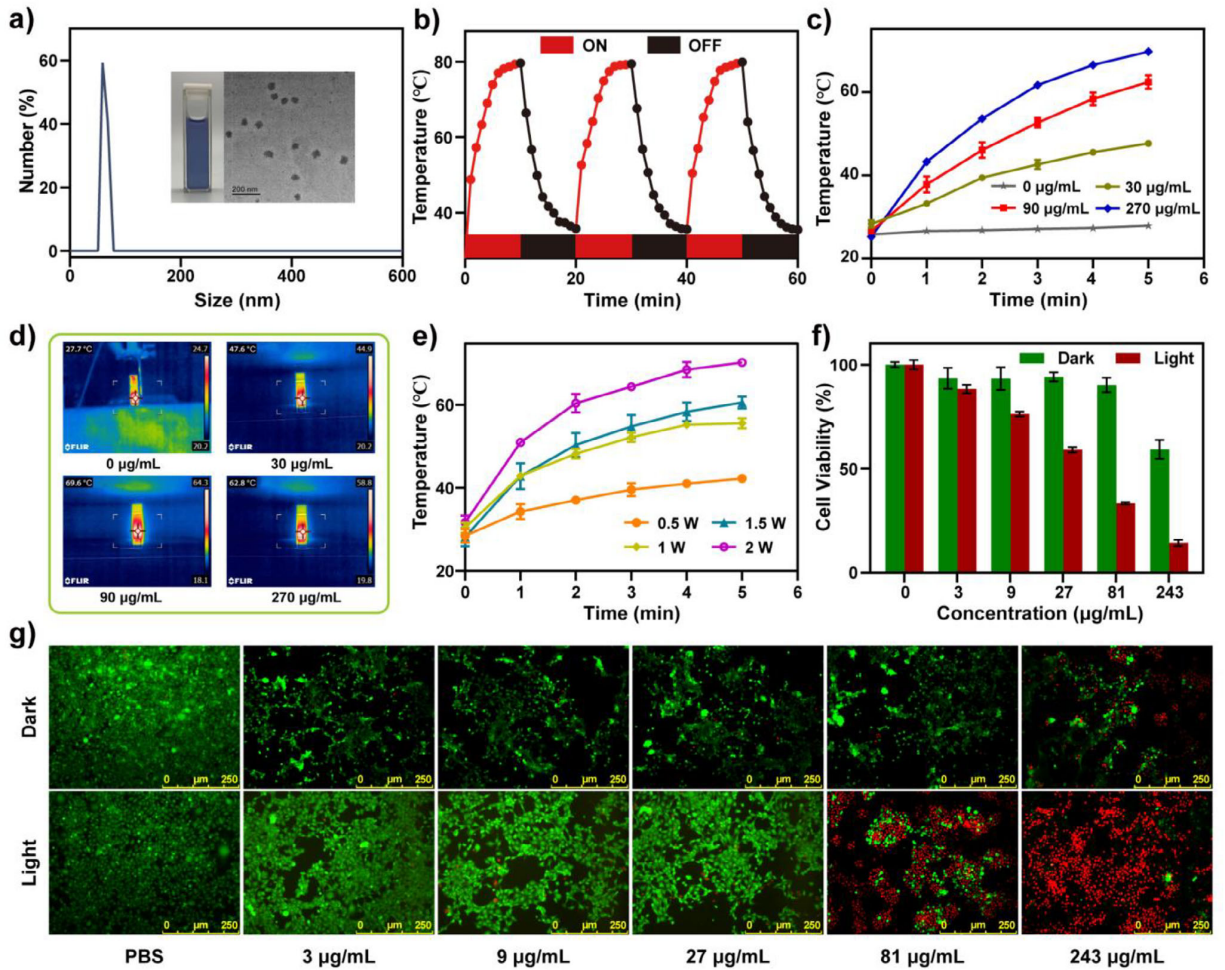
a) Dynamic light scattering spectrum of **7a‐NPs** in PBS solution. Insets: Photograph of **7a‐NPs** in PBS solution and TEM image of **7a‐NPs** (Scale: 200 nm); b) Temperature cycling curves of **7a‐NPs** with three cycles of light irradiation and natural cooling (808 nm, 2.0 W cm^−2^, *c* = 2 mg mL^−1^); c) Temperature increase curves of **7a‐NPs** at different concentrations (808 nm, 2.0 W cm^−2^); d) Thermal images of **7a‐NPs** at different concentrations after 5 min of light exposure (808 nm, 2.0 W cm^−2^); e) Temperature increase curves of **7a‐NPs** under different laser power irradiation (808 nm, *c* = 270 µg mL^−1^); f) Cell viability of 4T1 cells treated with **7a‐NPs** at different concentrations after exposure to dark and near‐infrared light (808 nm, 2.0 W cm^−2^, 5 min); g) Fluorescence images of cell viability using Calcein‐AM/PI staining after treatment of 4T1 cells with different concentrations of **7a‐NPs** (*c* = 0–243 µg mL^−1^) under dark conditions and near‐infrared light (808 nm, 2.0 W cm^−2^, 5 min) irradiation.

Based on the experimental results mentioned above, it can be inferred that the excited state of macrocyclic BODIPY derivatives **7(a–d)** exhibits weak RT and ISC capabilities, suggestive of strong NRT ability, such as photothermal conversion. Furthermore, compared with other macrocyclic BODIPY derivatives, the maximum absorption wavelength redshift of compound **7a** is the most significant, enabling excitation by lower energy light, reducing photodamage to biological tissues. To investigate the potential of **7a** as a photothermal agent, we employed DSPE‐mPEG_2000_ as an encapsulation matrix and successfully constructed **7a‐NPs**. The successful construction of **7a‐NPs** was confirmed using infrared spectroscopy (Figure S59). Compared to **7a** (which has good lipid solubility but poor water solubility), the **7a‐NPs** exhibit excellent water solubility, forming a homogeneous dark blue solution when dissolved in water (Figures 5 and S60). **7a‐NPs** exhibit absorption spectra similar to **7a** in the range of 700–900 nm, indicating that **7a‐NPs** also possess excellent near‐infrared light absorption capability (Figure S61). As shown in Figure 5a, transmission electron microscopic analysis revealed that **7a‐NPs** are spherical particles with an average diameter of approximately 55 ± 5 nm. Additionally, dynamic light scattering was used to determine the size distribution of **7a‐NPs** in aqueous solution, revealing an average particle size of approximately 63 nm and a zeta potential value of −3.79 mV.

Absorption spectra of freshly prepared and 4‐week‐old **7a‐NPs** solution were measured at room temperature, demonstrating the excellent physiological stability of **7a‐NPs** in PBS buffer (Figure S61). Subsequently, the photothermal conversion efficiency of **7a‐NPs** in PBS solution was investigated under laser irradiation at 808 nm (2.0 W cm^−2^). Firstly, the photothermal stability of **7a‐NPs** was validated through three cycles of laser irradiation‐induced heating and natural cooling experiments with a **7a‐NPs** concentration of 2 mg mL^−1^ and 808 nm excitation light power of 2 W cm^−2^. The results, as shown in Figure 5b, confirmed the excellent photothermal stability of the **7a‐NPs** (Figures S62–S64). Furthermore, the photothermal heating capacity of the **7a‐NPs** solutions at different concentrations were systematically investigated. As shown in Figure 5c,d, the solution temperature increased with increasing **7a‐NPs** concentration. At a concentration of 270 µg mL^−1^, the solution temperature reached 69.7 °C for **7a‐NPs**, while there was no significant change in temperature observed for a PBS solution (Figures S65–S68). In addition, the effect of light power on the photothermal conversion ability of **7a‐NPs** was investigated using different excitation power levels (0.5–2.0 W cm^−2^) of 808 nm light. As shown in Figure 5e, when the **7a‐NPs** concentration was 270 µg mL^−1^, the solution temperature increased with increasing excitation light power. At excitation light powers of 0.5, 1, 1.5, and 2 W cm^−2^, the solution temperatures reached 41.8 °C, 55.3 °C, 60.5 °C, and 70.1 °C, respectively (Figures S69–S72). Finally, calculations based on the natural cooling data of the **7a‐NPs** solution revealed a photothermal conversion efficiency of 44.6% at 808 nm (Figure S73).^[^ ^55^, ^56^ ^]^ Compared to some non‐BODIPY organic small molecule photothermal agents that can be excited by 808 nm light, the **7a‐NPs** exhibit higher PCE and stronger photothermal performance (as shown in Table S5 and Figure S74 for **ICG**, **NIR6**, **NIRb6**, **NIRb10**, **NIRb14**, **BTPETTQ**, **4‐Pf**, and **2TPE‐NDTA**). While structurally more complex non‐BODIPY organic small molecule photothermal agents, with more complex syntheses exhibit higher PCEs (i.e. **2TPE‐2NDTA**, **PQDI‐NP**, and **IR‐Y6** see Table S5 and Figure S74). In comparison to linear BODIPY photosensitizers, the macrocyclic BODIPY derivatives developed as part of this research exhibit enhanced PCEs (as seen for **A1** and **aza‐BODIPY‐mPEG** in Table S5 and Figure S74). Compared to some linear BODIPY derivatives that introduce special functional groups, the expansion of the light absorption range into the near‐infrared region for macrocyclic BODIPY derivatives is limited (as seen for **QLD‐BDP** and **CNTPA** in Table S5 and Figure S74). Additionally, the water solubility of macrocyclic BODIPY derivatives is lower than that of BODIPY photothermal agents containing glucose molecules (as seen for **AzuGIu‐BODIPY** in Table S5 and Figure S74). Therefore, the strategy of incorporating glucose molecules to enhance water solubility is worth exploration in future research. One previously developed strategy to enhance the PCE of BODIPY photosensitizers, involves introducing freely rotatable CF_3_ groups at the meso position of the BODIPY core. The linear BODIPY photosensitizers developed using this strategy have achieved a PCE exceeding 80%, a value which our system currently does not match (as shown by **tfm‐BDP** and **TCF_3_P** in Table S5 and Figure S74). However, combining the strategy of introducing freely rotatable CF_3_ groups and our research may result in systems with improved application prospects in the field of photothermal therapy. The **7a‐NPs** demonstrate excellent water solubility and photothermal conversion efficiency in photothermal conversion experiments. To assess the biocompatibility of **7a‐NPs**, the photothermal conversion performance of **7a‐NPs** was systematically investigated in different media. Compared to a **7a** solution dispersed in DMSO, the photothermal conversion performance of **7a‐NPs** dispersed in PBS is slightly reduced (Figures S75–S77). The photothermal conversion performance of **7a‐NPs** dispersed in serum and culture medium is consistent with that of **7a‐NPs** dispersed in PBS (Figure S78–S79). This suggests that **7a‐NPs** exhibit good stability and are compatible with different dispersing media. Subsequently, the biocompatibility and phototoxicity of **7a‐NPs** were further validated through CCK‐8 cell experiments. Under dark conditions, 4T1, HUVEC, 3T3, and HL‐7702 cells co‐incubated with **7a‐NPs** (81 µg mL^−1^) maintained a survival rate of over 80% (Figure S88). Furthermore, the survival rates of 4T1, HUVEC, 3T3, and HL‐7702 cells co‐incubated with **7a** (solvent: DMSO) were similar to those of DMSO alone. This indicates that **7a** has negligible cytotoxicity. Based on the survival rate of 4T1 cells, the IC_50_ value of **7a‐NPs** under dark conditions was calculated to be 326 µg mL^−1^. Subsequently, the photothermal efficacy of **7a‐NPs** was evaluated with a 4T1 tumor cell line under near‐infrared laser irradiation (808 nm, 2.0 W cm^−2^). Compared to the control group, **7a‐NPs** irradiated with laser exhibited a significant cytotoxic effect on 4T1 cells, and the cytotoxicity was directly proportional to the concentration of **7a‐NPs** (Figure 5f). At the same time, it can be seen from the calculation that the IC_50_ value of **7a‐NPs** under the illumination condition is 36.7 µg mL^−1^. Using IC_50_ values under dark and light conditions, the phototoxicity index of **7a‐NPs** was further calculated to be 8.88, exhibiting obvious phototoxicity potential. Furthermore, the cytotoxicity of **7a‐NPs** on cells under dark and light exposure was further evaluated using Calcein‐AM/PI staining (green for live cells, red for dead cells). As shown in Figure 5g, both PBS and **7a‐NPs** exhibit low cytotoxicity under dark conditions. However, under laser irradiation, cell death rates increase with higher sample concentrations. At a concentration of 81 µg mL^−1^ of **7a‐NPs**, the stained tumor cells displayed a strong red fluorescence, indicating a significant amount of cell death. This result confirms that **7a‐NPs** exhibit high cytotoxicity against tumor cells under laser irradiation, illustrating promising potential for their application in tumor cell eradication.

### In Vivo Tumor Therapy

Based on the aforementioned in vitro cell experimental results, we selected **7a‐NPs** as a photothermal agent for the treatment of 4T1 tumor‐bearing mice to explore the therapeutic efficacy in vivo. The hemolysis test results indicate that **7a‐NPs** do not cause red blood cell damage and have no negative impact on blood (Figure S89). Next, **7a‐NPs** were injected into mice through the tail vein, and the distribution of **7a‐NPs** in mice was detected using UV–vis‐NIR absorption spectroscopy after 6 h of culture. The results indicated that **7a‐NPs** could successfully enter various organs (liver, brain, kidney, heart) in mice through the blood (Figure S90). Subsequently, the biocompatibility and toxicity of the **7a‐NPs** were evaluated in mice. **7a‐NPs** were administered to mice at fixed doses (25 and 50 mg kg^−1^), and changes in the mice body weight were monitored over 21 days which confirmed that **7a‐NPs** do not affect the normal growth and development of mice (Figure S91). Results from blood biochemistry tests (after 21 days of culture) and mice organ histological analysis (after 1 and 3 days of culture) also confirmed that **7a‐NPs** do not have a negative impact on the normal growth and development of the mice (Figures S92 and S93). We then investigated the photothermal heating capabilities of **7a‐NPs** under different thicknesses of fat and muscle barriers by constructing a model that simulates the heating process. After 5 min of near‐infrared light irradiation (808 nm, 2.0 W cm^−2^), the maximum temperatures reached by solutions of **7a‐NPs** were 47.2 °C, 37.9 °C, and 28.8 °C for a fat thicknesses of 1–3 mm (Figures S94a and S95–S97). For a muscle thicknesses of 1–3 mm, the maximum temperatures reached by the solutions of **7a‐NPs** were 54.2 °C, 43.6 °C, and 41.2 °C (Figures S94b and S98–S100) under the same near‐infrared light irradiation conditions. These photothermal heating results demonstrate that **7a‐NPs** exhibits excellent photothermal conversion capabilities under different thicknesses of fat and muscle. **7a‐NPs** were then injected subcutaneously and intramuscularly into mice to investigate their photothermal heating capabilities in vivo. After 4 min of light irradiation (808 nm, 2.0 W cm^−2^), there was a positive correlation between the heating capabilities and the concentration of **7a‐NPs** in both subcutaneous and intramuscular regions of mice. At **7a‐NPs** concentrations of 30, 90, and 270 µg mL^−1^, the subcutaneous temperature in mice reached 49.7 °C, 56.8 °C, and 65.1 °C, respectively (Figures S94c and S101–S103). Similarly, at the same concentrations of **7a‐NPs**, the intramuscular temperature in mice reached 43.6 °C, 45.7 °C, and 54.0 °C, respectively (Figures S94d and S104–S106). These results indicate that the temperature achieved by low concentrations of **7a‐NPs** through photothermal conversion is sufficient to kill tumor cells in both subcutaneous and intramuscular regions of mice.

**Figure 6:**
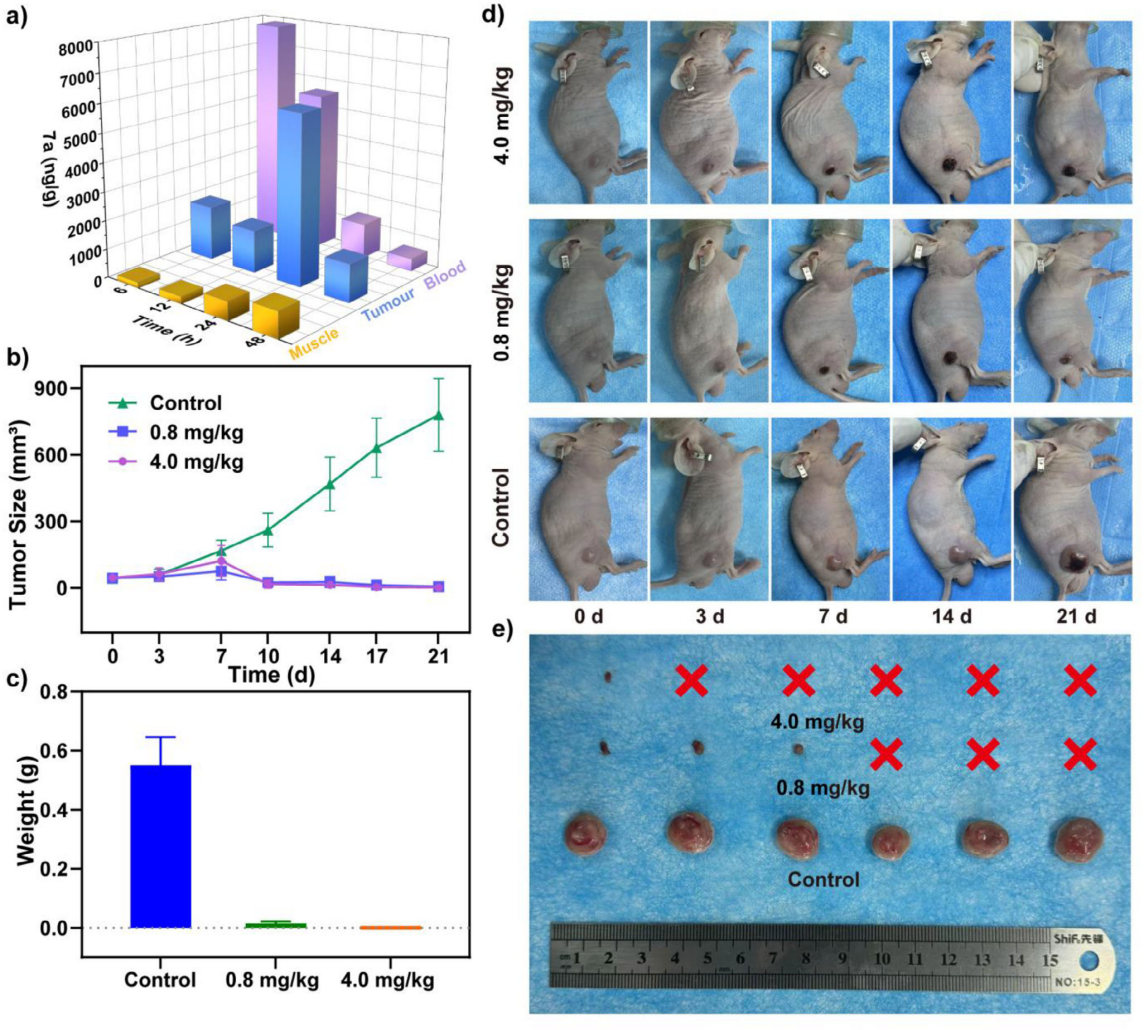
a) Schematic diagram of **7a** levels in blood, tumor, and muscle tissue of mice over time; b) Schematic diagram illustrating the changes in tumor volume during treatment; c) Schematic diagram illustrating the tumor mass after treatment completion; d) Schematic diagram illustrating the tumor photographs of mice during treatment; e) Schematic diagram illustrating the residual tumor after treatment completion, where “×” represents completely ablated tumors.

During the PTT of tumors, enrichment of **7a‐NPs** at the tumor site is key to affecting the efficacy of PTT. To determine whether **7a‐NPs** can enter the tumor site and enrich, we measured the content of **7a** in tumor, muscle, and blood (associated with PTT) of 4T1 tumor‐bearing mice cultured at different times after tail vein injection with **7a‐NPs**. The results indicated that **7a‐NPs** reached various parts of the mouse body through the blood after injection through the tail vein. With the extension of culture time, the content of **7a‐NPs** in the blood of mice gradually decreased, and the content of **7a‐NPs** in the tumor and muscle gradually increased (reaching the maximum concentration after 24 h) (Figure 6a). To investigate the PTT effect of **7a‐NPs** in mice, a randomized study was conducted with control groups (PBS + light), low concentration groups (0.8 mg kg^−1^ **7a‐NPs** + light), and high concentration groups (4.0 mg kg^−1^ **7a‐NPs** + light), each consisting of 6 mice, in a 4T1 tumor‐bearing mice model (with an average tumor volume of 40 mm^3^). After intravenous injection of PBS and **7a‐NPs** (100 µL) for 24 h, mice were subjected to 3 min of treatment using a 808 nm laser while controlling the local tumor temperature within the range of 43 °C–45 °C (by adjusting the laser power) to achieve an optimal tumor ablation temperature. For the low concentration group, the temperature reached 43 °C–45 °C within 2 min and was maintained for 1 min, while in the high concentration group, the temperature reached 43 °C–45 °C within 30 s and was maintained for 2.5 min. The PTT therapeutic effect of the **7a‐NPs** in mice was evaluated by monitoring the tumor growth in each group. As shown in Figure 6b, the “Control group” treated only with “PBS + light” failed to inhibit tumor growth (tumor volume increased 19‐fold), indicating that laser irradiation did not have a negative impact on tumor growth. The low concentration group (0.8 mg kg^−1^) treated with “**7a‐NPs** + light” intervention effectively suppressed tumor growth and exhibited tumor elimination capability. It is worth noting that the high concentration group (4.0 mg kg^−1^) treated with “**7a‐NPs** + light” intervention exhibited higher anti‐tumor activity and achieved almost complete tumor elimination. The tumor weights and photographs after treatment under different conditions, as well as the photographs of mice at different treatment time points, further confirm these conclusions (Figure 6c–e). In addition, the body weights of all mice in the different treatment groups were monitored during the tumor treatment process, confirming that **7a‐NPs** and light exposure did not affect the normal feeding, growth, and development of the mice (Figure S107). In addition, the destruction of the tumor surrounding tissue by photothermal therapy was investigated using a tissue section staining experiment. The results indicated that only the tissue covered by the light spot had light damage, and did not have a negative effect on the surrounding normal tissue (Figure S108). Therefore, the above experimental results confirm that **7a‐NPs** are a photothermal reagent with good biocompatibility, low toxicity and high tumor ablation efficiency, and exhibit significant application potential in the field of PTT therapy.

## Conclusion

In summary, we have designed and synthesized four macrocyclic BODIPY derivatives with stable cyclic structures. The construction of intramolecular D/A systems and introduction of the C_6_F_5_ electron‐withdrawing group promote intramolecular charge transfer, reduce the HOMO–LUMO gap, and redshift the maximum absorption wavelength of **7a** to 794 nm. The X‐ray crystal structure and theoretical calculations confirm that the distortion of the intramolecular cyclic structure leads to weaker ISC capability. Thanks to intramolecular charge transfer, low fluorescence, weak ISC capability, and the stable cyclic structure, **7a‐NPs** exhibit excellent photothermal stability and a high PCE of up to 44.6%. In vitro and in vivo experiments confirm the potential and practical value of **7a‐NPs** as photothermal agents. Our results demonstrate the feasibility of using a macrocyclization strategy to expand the light absorption range of photosensitizers and convert the RT process into an NRT process. Therefore, this study provides a new strategy for the development of near‐infrared BODIPY photothermal agents with high NRT capability.

## Experimental Section

Experimental details are available in the Supporting Information.

## Conflict of Interests

TDJ acts as an academic consultant for TQ as part of a guest professorship at SUST. All other authors declare that they have no conflict of interest.

## Supporting information



Supporting Information

Supporting Information

## Data Availability

The data that support the findings of this study are available in the Supporting Information of this article.

## References

[1] X. H. Liu , Y. H. Li , K. Y. Wang , Y. Y. Chen , M. W. Shi , X. Zhang , W. Pan , N. Li , B. Tang , Nano Lett. 2021, 21, 7862–7869. 34494442 10.1021/acs.nanolett.1c03089

[2] J. H. Zeng , M. Wu , S. Y. Lan , J. Li , X. L. Zhang , J. F. Liu , X. L. Liu , Z. Z. Wei , Y. Y. Zeng , J. Mater. Chem. B 2018, 6, 7889–7897. 32255034 10.1039/c8tb02079e

[3] Z. H. Lu , Y. Chen , D. Liu , X. Jiao , C. Liu , Y. K. Wang , Z. Z. Zhang , K. R. Jia , J. F. Gong , Z. M. Yang , L. Shen , Nat. Med. 2023, 29, 3022–3032. 38087112 10.1038/s41591-023-02655-3

[4] L. Montégut , C. López‐Otín , G. Kroemer , Molecular Cancer 2024, 23, 106. 38760832 10.1186/s12943-024-02020-z PMC11102267

[5] R. Mancusi , M. Monje , Nature 2023, 618, 467–479. 37316719 10.1038/s41586-023-05968-y PMC11146751

[6] M. Malvezzi , C. Santucci , P. Boffetta , G. Collatuzzo , F. Levi , C. L. Vecchia , E. Negri , Ann. Oncol. 2023, 34, 410–419. 36882139 10.1016/j.annonc.2023.01.010

[7] Y. J. Wang , C. R. Liu , C. Fang , Q. X. Peng , W. Qin , X. B. Yan , K. Zhang , Nano‐Micro Lett. 2025, 17, 30. 10.1007/s40820-024-01533-y PMC11442722 39347944

[8] K. R. Keshari , D. A. Heller , R. Boltyanskiy , H. Hricak , T. Magaldi , M. Overholtzer , Cancer Cell 2024, 42, 1138–1141. 38848719 10.1016/j.ccell.2024.04.013 PMC12282263

[9] T. X. Jin , D. Cheng , G. Y. Jiang , W. Q. Xing , P. W. Liu , B. Wang , W. P. Zhu , H. T. Sun , Z. R. Sun , Y. F. Xu , X. H. Qian , Bioact. Mater. 2022, 14, 42–51. 35310343 10.1016/j.bioactmat.2021.12.009 PMC8892148

[10] Y. D. Zhang , Z. Y. Yang , X. H. Zheng , L. Chen , Z. G. Xie , J. Mater. Chem. B 2020, 8, 5305–5311. 32453332 10.1039/d0tb00991a

[11] Z. L. Yan , M. Y. Wang , M. K. Shi , Y. He , Y. Zhang , S. H. Qiu , H. Yang , H. B. Chen , H. He , Z. Q. Guo , J. Mater. Chem. B 2020, 8, 6886–6897. 32323684 10.1039/d0tb00609b

[12] W. Shao , C. Yang , F. Y. Li , J. H. Wu , N. Wang , Q. Ding , J. Q. Gao , D. S. Ling , Nano‐Micro Lett. 2020, 12, 147. 10.1007/s40820-020-00474-6 PMC7770699 34138129

[13] Q. Wu , G. Chen , K. K. Gong , J. Wang , X. X. Ge , X. Q. Liu , S. J. Guo , F. Wang , Matter 2019, 1, 496–512.

[14] N. Song , L. Fu , Y. Liu , Y. Y. Li , L. Chen , X. Y. Wang , S. Liu , Z. G. Xie , Dyes Pigm. 2019, 162, 295–302.

[15] X. Liu , H. L. Su , W. Shi , Y. Liu , Y. N. Sun , D. T. Ge , Biomaterials 2018, 167, 177–190. 29571053 10.1016/j.biomaterials.2018.03.030

[16] T. K. Luo , K. Y. Ni , A. Culbert , G. X. Lan , Z. Li , X. M. Jiang , M. Kaufmann , W. B. Lin , J. Am. Chem. Soc. 2020, 142, 7334–7339. 32248686 10.1021/jacs.0c02129

[17] G. X. Lan , K. Y. Ni , S. S. Veroneau , X. Y. Feng , G. T. Nash , T. K. Luo , Z. W. Xu , W. B. Lin , J. Am. Chem. Soc. 2019, 141, 4204–4208. 30779556 10.1021/jacs.8b13804

[18] L. Zhang , S. B. Wang , Y. Zhou , C. Wang , X. Z. Zhang , H. X. Deng , Angew. Chem. Int. Ed. 2019, 58, 14213–14218. 10.1002/anie.201909020 31347259

[19] H. Wang , X. Z. Yang , W. Shao , S. C. Chen , J. F. Xie , X. D. Zhang , J. Wang , Y. Xie , J. Am. Chem. Soc. 2015, 137, 11376–11382. 26284535 10.1021/jacs.5b06025

[20] L. N. Feng , C. B. Li , L. X. Liu , Z. Y. Wang , Z. H. Chen , J. Yu , W. W. Ji , G. Y. Jiang , P. F. Zhang , J. G. Wang , B. Z. Tang , ACS Nano 2022, 16, 4162–4174. 35230081 10.1021/acsnano.1c10019

[21] Z. J. Zhang , W. H. Xu , M. M. Kang , H. F. Wen , H. Guo , P. F. Zhang , L. Xi , K. Li , L. Wang , D. Wang , B. Z. Tang , Adv. Mater. 2020, 32, e2003210. 32696561 10.1002/adma.202003210

[22] D. Y. Yan , M. Wang , Q. Wu , N. Niu , M. Li , R. X. Song , J. Rao , M. M. Kang , Z. J. Zhang , F. F. Zhou , D. Wang , B. Z. Tang , Angew. Chem. Int. Ed. 2022, 61, e202202614. 10.1002/anie.202202614 35344252

[23] X. F. Meng , B. Y. Zhang , Y. Yi , H. Cheng , B. M. Wang , Y. Y. Liu , T. Gong , W. Yang , Y. F. Yao , H. Wang , W. B. Bu , Nano Lett. 2020, 20, 2522–2529. 32208714 10.1021/acs.nanolett.9b05267

[24] Y. S. Liu , Y. Li , S. Koo , Y. Sun , Y. X. Liu , X. Liu , Y. N. Pan , Z. Y. Zhang , M. X. Du , S. Y. Lu , X. Qiao , J. F. Gao , X. B. Wang , Z. X. Deng , X. L. Meng , Y. L. Xiao , J. S. Kim , X. C. Hong , Chem. Rev. 2022, 122, 209–268. 34664951 10.1021/acs.chemrev.1c00553

[25] L. Taiariol , C. Chaix , C. Farre , E. Moreau , Chem. Rev. 2022, 122, 340–384. 34705429 10.1021/acs.chemrev.1c00484

[26] C. N. Li , W. H. Lin , S. Liu , W. Zhang , Z. G. Xie , J. Mater. Chem. B 2019, 7, 4655–4660. 31364670 10.1039/c9tb00752k

[27] Y. C. Yan , J. W. Chen , Z. J. Yang , X. Zhang , Z. Liu , J. L. Hua , J. Mater. Chem. B 2018, 6, 7420–7426. 32254743 10.1039/c8tb01750f

[28] C. Ma , T. Zhang , Z. G. Xie , J. Mater. Chem. B 2021, 9, 7318–7327. 34355720 10.1039/d1tb00855b

[29] F. R. Wu , Y. J. Liu , Y. Wu , D. W. Song , J. W. Qian , B. S. Zhu , J. Mater. Chem. B 2020, 8, 2128–2138. 32073096 10.1039/c9tb02646k

[30] P. H. Cheng , K. Y. Pu , ACS Appl. Mater. Interfaces 2020, 12, 5286–5299. 31730329 10.1021/acsami.9b15064

[31] S. Ayan , G. Gunaydin , N. Yesilgul‐Mehmetcik , M. E. Gedik , O. Seven , E. U. Akkaya , Chem. Commun. 2020, 56, 14793–14796. 10.1039/d0cc06031c 33196713

[32] K. Y. Wang , Y. N. Xiang , W. Pan , H. Y. Wang , N. Li , B. Tang , Chem. Sci. 2020, 11, 8055–8072. 34123080 10.1039/d0sc03173a PMC8163445

[33] X. Z. Zhao , S. R. Long , M. L. Li , J. F. Cao , Y. C. Li , L. Y. Guo , W. Sun , J. J. Du , J. L. Fan , X. J. Peng , J. Am. Chem. Soc. 2020, 142, 1510–1517. 31880443 10.1021/jacs.9b11800

[34] J. W. Zhu , J. H. Zou , J. Zhang , Y. Sun , X. C. Dong , Q. Zhang , J. Mater. Chem. B 2019, 7, 3303–3309.

[35] N. Song , Y. Y. Li , L. Chen , X. L. Hu , Z. G. Xie , J. Mater. Chem. B 2019, 7, 3976–3981.

[36] Q. Yu , T. C. Huang , C. Liu , M. L. Zhao , M. J. Xie , G. Li , S. J. Liu , W. Huang , Q. Zhao , Chem. Sci. 2019, 10, 9091–9098. 31827751 10.1039/c9sc03161h PMC6889832

[37] K. X. Lv , H. M. Lin , F. Y. Qu , Chem. Eng. J. 2020, 392, 124555.

[38] J. C. Li , D. Cui , Y. Y. Jiang , J. G. Huang , P. H. Cheng , K. Y. Pu , Adv. Mater. 2019, 31, e1905091. 31566279 10.1002/adma.201905091

[39] Z. M. Wang , X. Zhen , P. K. Upputuri , Y. Y. Jiang , J. W. Lau , M. Pramanik , K. Y. Pu , B. G. Xing , ACS Nano 2019, 13, 5816–5825. 31034202 10.1021/acsnano.9b01411

[40] Y. Cao , Y. N. Wu , G. N. Wang , J. W. Yi , C. L. Yu , Y. X. Huang , L. G. Sun , Y. L. Bao , Y. X. Li , J. Mater. Chem. B 2017, 5, 5479–5487. 32264088 10.1039/c7tb01264k

[41] H. S. Jung , J. Y. Han , H. Shi , S. Koo , H. Singh , H. J. Kim , J. L. Sessler , J. Y. Lee , J. H. Kim , J. S. Kim , J. Am. Chem. Soc. 2017, 139, 7595–7602. 28459562 10.1021/jacs.7b02396 PMC5772932

[42] Z. Zhou , J. Zhou , L. Gai , A. Yuan , Z. Shen , Chem. Commun. 2017, 53, 6621–6624. 10.1039/c7cc02918g 28585640

[43] D. M. Xi , M. Xiao , J. F. Cao , L. Y. Zhao , N. Xu , S. R. Long , J. L. Fan , K. Shao , W. Sun , X. H. Yan , X. J. Peng , Adv. Mater. 2020, 32, e1907855. 32022978 10.1002/adma.201907855

[44] W. B. Hu , X. F. Miao , H. J. Tao , A. Baev , C. Ren , Q. L. Fan , T. C. He , W. Huang , P. N. Prasad , ACS Nano 2019, 13, 12006–12014. 31518102 10.1021/acsnano.9b06208

[45] W. Zhang , W. H. Lin , X. Wang , C. N. Li , S. Liu , Z. G. Xie , ACS Appl. Mater. Interfaces 2019, 11, 278–287. 30520633 10.1021/acsami.8b17922

[46] W. Zhang , W. H. Lin , C. N. Li , S. Liu , X. L. Hu , Z. G. Xie , ACS Appl. Mater. Interfaces 2019, 11, 32720–32728. 31433153 10.1021/acsami.9b10713

[47] D. P. Chen , Y. Y. Tang , J. W. Zhu , J. J. Zhang , X. J. Song , W. J. Wang , J. J. Shao , W. Huang , P. Chen , X. C. Dong , Biomaterials 2019, 221, 119422. 31437723 10.1016/j.biomaterials.2019.119422

[48] Z. Q. Guo , H. He , Y. Zhang , J. M. Rao , T. Yang , T. Li , L. Wang , M. K. Shi , M. Y. Wang , S. H. Qiu , X. Song , H. T. Ke , H. B. Chen , Adv. Mater. 2021, 33, e2004225. 33270303 10.1002/adma.202004225

[49] D. M. Xi , M. Xiao , J. F. Cao , L. Y. Zhao , N. Xu , S. R. Long , J. L. Fan , K. Shao , W. Sun , X. H. Yan , X. J. Peng , Adv. Mater. 2020, 32, 1907855. 10.1002/adma.201907855 32022978

[50] C. Y. Li , G. C. Chen , Y. J. Zhang , F. Wu , Q. B. Wang , J. Am. Chem. Soc. 2020, 142, 14789–14804. 32786771 10.1021/jacs.0c07022

[51] J. C. Chen , H. G. Nie , Y. T. Rao , L. Xu , M. B. Zhou , B. S. Yin , J. X. Song , A. Osuka , Chem Asian J 2024, 19, e202400029. 38458988 10.1002/asia.202400029

[52] K. L. Liu , Z. Q. Jiang , R. A. Lalancette , X. Y. Tang , F. Jakle , J. Am. Chem. Soc. 2022, 144, 18908–18917. 36194812 10.1021/jacs.2c06538

[53] Z. J. Wang , L. Huang , Y. X. Yan , A. M. El‐Zohry , A. Toffo‐letti , J. Z. Zhao , A. Barbon , B. Dick , O. F. Mohammed , G. Han , Angew. Chem. Int. Ed. 2020, 59, 16114–16121. 10.1002/anie.202005269 PMC7540422 32449273

[54] X. Y. Dong , X. Y. Dai , Y. M. Zhang , X. F. Xu , Y. Liu , Adv. Sci. 2022, 9, e2201962. 10.1002/advs.202201962 PMC9376817 35713271

[55] Q. W. Tian , F. R. Jiang , R. J. Zou , Q. Liu , Z. G. Chen , M. F. Zhu , S. P. Yang , J. L. Wang , J. H. Wang , J. Q. Hu , ACS Nano 2011, 5, 9761–9771. 22059851 10.1021/nn203293t

[56] D. K. Roper , W. Ahn , M. Hoepfner , J. Phys. Chem. C 2007, 111, 3636–3641. 10.1021/jp064341w PMC2583113 19011696

