# Supplementary material for: Near‐Infrared Conjugated Macrocyclic BODIPYs for Photothermal Cancer Therapy
Source: Angew Chem Int Ed Engl. 2025 Sep 27;64(47):e202511125. doi: 10.1002/anie.202511125 (PMC12624305; doi:10.1002/anie.202511125)
Supplement: Supplementary file 1 — Supporting Information [file ANIE-64-e202511125-s001.doc]

**Supporting Information**

**Near-Infrared Conjugated Macrocyclic BODIPYs for Photothermal Cancer Therapy**

Fei Cheng^a^, Taotao Qiang^a, *^, Mingli Li^a^, Ruilong Li^a^, Zhigao Wang^c^, Tony D. James^b *^

[a] College of Bioresources and Materials Engineering, Shaanxi Collaborative Innovation Center of Industrial Auxiliary Chemistry & Technology, Shaanxi University of Science & Technology, Xi'an, 710021, China.

[b] Department of Chemistry, University of Bath, Bath, BA27AY, UK.

[c] College of Pharmacy, Xi'an Medical University, Xi'an, 710021, China.

## Instrumentation and Materials

All reactions involving air-sensitive intermediates or catalysts were carried out under an atmosphere of pre-purified nitrogen using Schlenk techniques. Toluene, *p*-xylene and DCM were distilled from CaH_2_ and degassed via several freeze-pump-thaw cycles. All other chemicals were purchased from commercial sources and directly used without further purification unless otherwise noted. NMR data were acquired at ambient temperature unless otherwise noted. 600 MHz ^1^H and 151 MHz ^13^C NMR data were recorded on a 600 MHz Bruker AVANCE spectrometer; ^1^H and ^13^C NMR spectra were referenced internally to solvent signals (CDCl_3_: 7.26 ppm for ^1^H NMR, 77.23 ppm for ^13^C NMR). High-resolution MALDI-TOF MS measurements were performed on a Bruker Ultraflextreme in reflectron mode with delayed extraction. The geometries of the ground state structures of BDP-based molecules were fully optimized without any structural constraints by density functional theory (DFT) in Gaussian 16 package. The frequency calculations were performed by the same level of theory to confirm that the optimized ground-state geometry was a stable structure. Time-dependent DFT (TD-DFT) calculations were carried out for the vertical excitation energies of both singlet and triplet excited states. The density functional PBE0 and basis set 6-31G(d, p) were employed in the calculation. The spin-orbit coupling matrix elements were calculated by TD-DFT calculation as implemented in ORCA program. Red phosphorus was used for calibration. UV-visible absorption data were acquired on a Varian Cary 5000 UV-Vis/NIR spectrophotometer. The fluorescence data was measured using an Edinburgh Instruments FLS1000 spectrophotometer. Absolute quantum yields (*Φ_F_*) were measured on the Hamamatsu C11347-11 Quantaurus-QY spectrometer. TG measurements were conducted with a Shimadzu thermodynamic analysis system-DTG-60H (Shimadzu Corporation, Japan) at a heating rate of 10 °C/min under nitrogen atmosphere) using about 3 mg of sample.

**Synthesis and Molecular Structure Characterization Data**

**Synthesis of 1a**^S1^: Pentafluorobenzaldehyde (5.0 g, 25.5 mmol) and pyrrole (51 g, 0.765 mol) were added into a dry 100 mL three-mouth round-bottom flask, and stirred at room temperature for 20 min under the protection of nitrogen, away from light. Trifluoroacetic acid (TFA, 0.2 mL) was added, and the reaction mixture was stirred 5 min at room temperature. After the reaction was complete, the raw materials were quenched with saturated sodium bicarbonate solution, dichloromethane was extracted, the organic phase was dried by anhydrous sodium sulfate, and the methylene chloride and pyrrole were recovered by rotary evaporation. The residue was purified by column chromatography (silica gel) with CH_2_Cl_2_/petroleum ether (v/v = 1:3) as eluent to provide **1a** (4.55 g, 57.2%). ^1^H NMR (600 MHz, CDCl_3_): δ = 8.15 (s, 2H), 6.74 (d, *J* = 2.4 Hz, 2H), 6.16 (d, *J* = 3.0 Hz, 2H), 6.02 (s, 2H), 5.90 (s, 1H) ppm.

**Synthesis of 1b** ^S2^: 4-Fluorobenzaldehyde (5.0 g, 40.3 mmol) and pyrrole (81 g, 1.21 mol) were added into a dry 100 mL three-mouth round-bottom flask, and stirred at room temperature for 20 min under the protection of nitrogen, away from light. Trifluoroacetic acid (TFA, 0.2 mL) was added, and the reaction mixture was stirred 5 min at room temperature. After the reaction was complete, the raw materials were quenched with saturated sodium bicarbonate solution, dichloromethane was extracted, the organic phase was dried by anhydrous sodium sulfate, and the methylene chloride and pyrrole were recovered by rotary evaporation. The residue was purified by column chromatography (silica gel) with CH_2_Cl_2_/petroleum ether (v/v = 1:2) as eluent to provide **1b** (5.35 g, 55.3%). ^1^H NMR (600 MHz, CDCl_3_): δ = 7.90 (s, 2H), 7.20-7.15 (m, 2H), 7.03-6.99 (m, 2H), 6.71-6.70 (m, 2H), 6.1-6.17 (m, 2H), 5.91-5.89 (m, 2H), 5.45 (s, 1H) ppm.

**Synthesis of 1c**^S3^: Mesitaldehyde (5.0 g, 33.8 mmol) and pyrrole (68 g, 1.01 mol) were added into a dry 100 mL three-mouth round-bottom flask, and stirred at room temperature for 20 min under the protection of nitrogen, away from light. Trifluoroacetic acid (TFA, 0.2 mL) was added, and the reaction mixture was stirred 1 h at room temperature. After the reaction was complete, the raw materials were quenched with saturated sodium bicarbonate solution, dichloromethane was extracted, the organic phase was dried by anhydrous sodium sulfate, and the methylene chloride and pyrrole were recovered by rotary evaporation. The residue was purified by column chromatography (silica gel) with CH_2_Cl_2_/petroleum ether (v/v = 1:2) as eluent to provide **1c** (4.5 g, 50.4%). ^1^H NMR (600 MHz, CDCl_3_): δ = 7.94 (s, 2H), 6.87 (s, 2H), 6.67-6.66 (m, 2H), 6.19-6.17 (m, 2H), 6.04-5.99 (m, 2H), 5.93 (s, 1H), 2.28 (s, 3H), 2.07 (s, 6H) ppm.

**Synthesis of 1d** ^S4^: 2,4,6-trimethoxybenzaldehyde (5.0 g, 25.5 mmol) and pyrrole (51 g, 0.765 mol) were added into a dry 100 mL three-mouth round-bottom flask, and stirred at room temperature for 20 min under the protection of nitrogen, away from light. Trifluoroacetic acid (TFA, 0.2 mL) was added, and the reaction mixture was stirred 5 min at room temperature. After the reaction was complete, the raw materials were quenched with saturated sodium bicarbonate solution, dichloromethane was extracted, the organic phase was dried by anhydrous sodium sulfate, and the methylene chloride and pyrrole were recovered by rotary evaporation. The residue was purified by column chromatography (silica gel) with CH_2_Cl_2_/petroleum ether (v/v = 3:2) as eluent to provide **1d** (3.9 g, 48.9%). ^1^H NMR (600 MHz, CDCl_3_): δ = 8.50 (s, 2H), 6.65-6.64 (m, 2H), 6.22 (s, 2H), 6.12-6.09 (m, 2H), 6.09 (s, 1H), 5.93-5.92 (m, 2H), 3.82 (s, 3H), 3.74 (s, 6H) ppm.

**Synthesis of 2a**^S5^: **1a** (1.0 g, 3.2 mmol) and 2,3-dichloro-5,6-dicyanobenzoquinone (DDQ, 0.8 g, 3.5 mmol) were added to a round-bottomed flask containing CH_2_Cl_2_ (50 mL), and the reaction mixture was stirred 1 h at room temperature. When the reaction is complete, the excess DDQ is removed with alumina. The organic layer was collected and evaporated under vacuum. The residue was dissolved in 1, 2-dichloroethane (100 mL) and stirred under argon at 50 °C for 30 min. Then Et_3_N (1.8 mL, 12.8 mmol) and BF_3_∙Et_2_O (2.4 mL, 19.2 mmol) were added dropwise, and the reaction mixture was stirred under argon at 85 °C for another 4 hours. Then it was washed with water, the organic layer was dried over Na_2_SO_4_, filtered and the solvent was evaporated under reduced pressure. The residue was purified by column chromatography (silica gel) with CH_2_Cl_2_/petroleum ether (v/v = 1:1) as eluent to provide **2a** (760 mg, 66.3%). ^1^H NMR (600 MHz, CDCl_3_): δ = 7.99 (s, 2H), 6.81 (d, *J* = 4.2 Hz, 2H), 6.58-6.57 (m, 2H) ppm; UV/Vis (CH_2_Cl_2_): λ_max_ (*ε* [M^-1^cm^-1^]) = 519 (78000) nm.

**Synthesis of 2b** ^S6^: **1b** (1.0 g, 4.2 mmol) and 2,3-dichloro-5,6-dicyanobenzoquinone (DDQ, 1.04 g, 4.6 mmol) were added to a round-bottomed flask containing CH_2_Cl_2_ (50 mL), and the reaction mixture was stirred 1 h at room temperature. When the reaction is complete, the excess DDQ is removed with alumina. The organic layer was collected and evaporated under vacuum. The residue was dissolved in 1, 2-dichloroethane (100 mL) and stirred under argon at 50 °C for 30 min. Then Et_3_N (2.4 mL, 16.8 mmol) and BF_3_∙Et_2_O (3.2 mL, 25.2 mmol) were added dropwise, and the reaction mixture was stirred under argon at 85 °C for another 4 hours. Then it was washed with water, the organic layer was dried over Na_2_SO_4_, filtered and the solvent was evaporated under reduced pressure. The residue was purified by column chromatography (silica gel) with CH_2_Cl_2_/petroleum ether (v/v = 4:3) as eluent to provide **2b** (810 mg, 67.5%). ^1^H NMR (600 MHz, CDCl_3_): δ = 7.95 (s, 2H), 7.60-7.56 (m, 2H), 7.23 (d, *J* = 8.4 Hz, 2H), 6.92 (d, *J* = 4.2 Hz, 2H), 6.57-6.56 (m, 2H) ppm; UV/Vis (CH_2_Cl_2_): λ_max_ (*ε* [M^-1^cm^-1^]) = 501 (78000) nm.

**Synthesis of 2c** ^S7^: **1c** (1.0 g, 3.8 mmol) and 2,3-dichloro-5,6-dicyanobenzoquinone (DDQ, 0.95 g, 4.2 mmol) were added to a round-bottomed flask containing CH_2_Cl_2_ (50 mL), and the reaction mixture was stirred 1 h at room temperature. When the reaction is complete, the excess DDQ is removed with alumina. The organic layer was collected and evaporated under vacuum. The residue was dissolved in 1, 2-dichloroethane (100 mL) and stirred under argon at 50 °C for 30 min. Then Et_3_N (2.2 mL, 15.2 mmol) and BF_3_∙Et_2_O (2.9 mL, 22.8 mmol) were added dropwise, and the reaction mixture was stirred under argon at 85 °C for another 4 hours. Then it was washed with water, the organic layer was dried over Na_2_SO_4_, filtered and the solvent was evaporated under reduced pressure. The residue was purified by column chromatography (silica gel) with CH_2_Cl_2_/petroleum ether (v/v = 4:3) as eluent to provide **2c** (787 mg, 66.8%). ^1^H NMR (600 MHz, CDCl_3_): δ = 7.91 (s, 2H), 6.95 (s, 2H), 6.68 (d, *J* = 4.2 Hz, 2H), 6.47-6.46 (m, 2H), 2.36 (s, 3H), 2.10 (s, 6H) ppm; UV/Vis (CH_2_Cl_2_): λ_max_ (*ε* [M^-1^cm^-1^]) = 501 (90000) nm.

**Synthesis of 2d** ^S8^: **1d** (1.0 g, 3.2 mmol) and 2,3-dichloro-5,6-dicyanobenzoquinone (DDQ, 0.8 g, 3.5 mmol) were added to a round-bottomed flask containing CH_2_Cl_2_ (50 mL), and the reaction mixture was stirred 1 h at room temperature. When the reaction is complete, the excess DDQ is removed with alumina. The organic layer was collected and evaporated under vacuum. The residue was dissolved in 1, 2-dichloroethane (100 mL) and stirred under argon at 50 °C for 30 min. Then Et_3_N (1.8 mL, 12.8 mmol) and BF_3_∙Et_2_O (2.4 mL, 19.2 mmol) were added dropwise, and the reaction mixture was stirred under argon at 85 °C for another 4 hours. Then it was washed with water, the organic layer was dried over Na_2_SO_4_, filtered and the solvent was evaporated under reduced pressure. The residue was purified by column chromatography (silica gel) with CH_2_Cl_2_/petroleum ether (v/v = 3:1) as eluent to provide **2d** (735 mg, 64.2%). ^1^H NMR (600 MHz, CDCl_3_): δ = 7.84 (s, 2H), 6.76 (d, *J* = 4.2 Hz, 2H), 6.43-6.42 (m, 2H), 6.21 (s, 2H), 3.89 (s, 3H), 3.67 (s, 6H) ppm; UV/Vis (CH_2_Cl_2_): λ_max_ (*ε* [M^-1^cm^-1^]) = 505 (88000) nm.

**Synthesis of 3a**: To a solution of **1a** (1.0 g, 3.2 mmol) in THF (100 mL) was added dropwise a solution of NBS (0.57 g, 3.2 mmol) in THF (20 mL) at -78 °C. The mixture was stirred at -78 °C for 30 min. 2,3-dichloro-5,6-dicyanobenzoquinone (DDQ, 0.8 g, 3.5 mmol) was added and stirring was continued for another 1 hour at room temperature. When the reaction is complete, the excess DDQ is removed with alumina. The organic layer was collected and evaporated under vacuum. The residue was dissolved in 1, 2-dichloroethane (100 mL) and stirred under argon at 50 °C for 30 min. Then Et_3_N (1.8 mL, 12.8 mmol) and BF_3_∙Et_2_O (2.4 mL, 19.2 mmol) were added dropwise, and the reaction mixture was stirred under argon at 85 °C for another 4 hours. Then it was washed with water, the organic layer was dried over Na_2_SO_4_, filtered and the solvent was evaporated under reduced pressure. The residue was purified by column chromatography (silica gel) with CH_2_Cl_2_/petroleum ether (v/v = 1:1) as eluent to provide **3a** (551 mg, 39.5%). ^1^H NMR (600 MHz, CDCl_3_): δ = 7.99 (s, 1H), 6.81 (d, *J* = 4.2 Hz, 1H), 6.71 (d, *J* = 4.2 Hz, 1H), 6.60-6.59 (m, 1H), 6.55 (d, *J* = 4.2 Hz, 1H) ppm.

**Synthesis of 3b**: To a solution of **1b** (1.0 g, 4.2 mmol) in THF (100 mL) was added dropwise a solution of NBS (0.75 g, 4.2 mmol) in THF (20 mL) at -78 °C. The mixture was stirred at -78 °C for 30 min. 2,3-dichloro-5,6-dicyanobenzoquinone (DDQ, 1.04 g, 4.6 mmol) was added and stirring was continued for another 1 hour at room temperature. When the reaction is complete, the excess DDQ is removed with alumina. The organic layer was collected and evaporated under vacuum. The residue was dissolved in 1, 2-dichloroethane (100 mL) and stirred under argon at 50 °C for 30 min. Then Et_3_N (2.4 mL, 16.8 mmol) and BF_3_∙Et_2_O (3.2 mL, 25.2 mmol) were added dropwise, and the reaction mixture was stirred under argon at 85 °C for another 4 hours. Then it was washed with water, the organic layer was dried over Na_2_SO_4_, filtered and the solvent was evaporated under reduced pressure. The residue was purified by column chromatography (silica gel) with CH_2_Cl_2_/petroleum ether (v/v = 4:3) as eluent to provide **3b** (559 mg, 36.6%). ^1^H NMR (600 MHz, CDCl_3_): δ = 7.97 (s, 1H), 7.56-7.51 (m, 2H), 7.25-7.22 (m, 2H), 6.89 (d, *J* = 4.2 Hz, 1H), 6.80 (d, *J* = 4.2 Hz, 1H), 6.60-6.57 (m, 1H), 6.54 (d, *J* = 4.2 Hz, 1H) ppm.

**Synthesis of 3c**: To a solution of **1c** (1.0 g, 3.8 mmol) in THF (100 mL) was added dropwise a solution of NBS (0.68 g, 3.8 mmol) in THF (20 mL) at -78 °C. The mixture was stirred at -78 °C for 30 min. 2,3-dichloro-5,6-dicyanobenzoquinone (DDQ, 0.95 g, 4.2 mmol) was added and stirring was continued for another 1 hour at room temperature. When the reaction is complete, the excess DDQ is removed with alumina. The organic layer was collected and evaporated under vacuum. The residue was dissolved in 1, 2-dichloroethane (100 mL) and stirred under argon at 50 °C for 30 min. Then Et_3_N (2.2 mL, 15.2 mmol) and BF_3_∙Et_2_O (2.9 mL, 22.8 mmol) were added dropwise, and the reaction mixture was stirred under argon at 85 °C for another 4 hours. Then it was washed with water, the organic layer was dried over Na_2_SO_4_, filtered and the solvent was evaporated under reduced pressure. The residue was purified by column chromatography (silica gel) with CH_2_Cl_2_/petroleum ether (v/v = 4:3) as eluent to provide **3c** (522 mg, 35.4%). ^1^H NMR (600 MHz, CDCl_3_): δ = 7.93 (s, 1H), 6.95 (s, 2H), 6.68 (d, *J* = 4.2 Hz, 1H), 6.56 (d, *J* = 4.2 Hz, 1H), 6.50-3.49 (m, 1H), 6.44 (d, *J* = 4.2 Hz, 1H), 2.36 (s, 3H), 2.09 (s, 6H) ppm.

**Synthesis of 3d**: To a solution of **1d** (1.0 g, 3.2 mmol) in THF (100 mL) was added dropwise a solution of NBS (0.57 g, 3.2 mmol) in THF (20 mL) at -78 °C. The mixture was stirred at -78 °C for 30 min. 2,3-dichloro-5,6-dicyanobenzoquinone (DDQ, 0.8 g, 3.5 mmol) was added and stirring was continued for another 1 hour at room temperature. When the reaction is complete, the excess DDQ is removed with alumina. The organic layer was collected and evaporated under vacuum. The residue was dissolved in 1, 2-dichloroethane (100 mL) and stirred under argon at 50 °C for 30 min. Then Et_3_N (1.8 mL, 12.8 mmol) and BF_3_∙Et_2_O (2.4 mL, 19.2 mmol) were added dropwise, and the reaction mixture was stirred under argon at 85 °C for another 4 hours. Then it was washed with water, the organic layer was dried over Na_2_SO_4_, filtered and the solvent was evaporated under reduced pressure. The residue was purified by column chromatography (silica gel) with CH_2_Cl_2_/petroleum ether (v/v = 3:1) as eluent to provide **3d** (575 mg, 41.2%). ^1^H NMR (600 MHz, CDCl_3_): δ = 7.86 (s, 1H), 6.76 (d, *J* = 4.2 Hz, 1H), 6.65 (d, *J* = 4.2 Hz, 1H), 6.45-6.44 (m, 1H), 6.40 (d, *J* = 4.2 Hz, 1H), 6.20 (s, 2H), 3.89 (s, 3H), 3.67 (s, 6H) ppm.

**Synthesis of 4a**^S9^: To a solution of **1a** (1.0 g, 3.2 mmol) in THF (100 mL) was added dropwise a solution of NBS (1.14 g, 6.4 mmol) in THF (20 mL) at -78 °C. The mixture was stirred at -78 °C for 30 min. 2,3-dichloro-5,6-dicyanobenzoquinone (DDQ, 0.8 g, 3.5 mmol) was added and stirring was continued for another 1 hour at room temperature. When the reaction is complete, the excess DDQ is removed with alumina. The organic layer was collected and evaporated under vacuum. The residue was dissolved in 1, 2-dichloroethane (100 mL) and stirred under argon at 50 °C for 30 min. Then Et_3_N (1.8 mL, 12.8 mmol) and BF_3_∙Et_2_O (2.4 mL, 19.2 mmol) were added dropwise, and the reaction mixture was stirred under argon at 85 °C for another 4 hours. Then it was washed with water, the organic layer was dried over Na_2_SO_4_, filtered and the solvent was evaporated under reduced pressure. The residue was purified by column chromatography (silica gel) with CH_2_Cl_2_/petroleum ether (v/v = 3:4) as eluent to provide **4a** (820 mg, 49.8%). ^1^H NMR (600 MHz, CDCl_3_): δ = 6.69 (d, *J* = 4.2 Hz, 2H), 6.57 (d, *J* = 4.2 Hz, 2H) ppm.

**Synthesis of 4b** ^S10^: To a solution of **1b** (1.0 g, 4.2 mmol) in THF (100 mL) was added dropwise a solution of NBS (1.5 g, 8.4 mmol) in THF (20 mL) at -78 °C. The mixture was stirred at -78 °C for 30 min. 2,3-dichloro-5,6-dicyanobenzoquinone (DDQ, 1.04 g, 4.6 mmol) was added and stirring was continued for another 1 hour at room temperature. When the reaction is complete, the excess DDQ is removed with alumina. The organic layer was collected and evaporated under vacuum. The residue was dissolved in 1, 2-dichloroethane (100 mL) and stirred under argon at 50 °C for 30 min. Then Et_3_N (2.4 mL, 16.8 mmol) and BF_3_∙Et_2_O (3.2 mL, 25.2 mmol) were added dropwise, and the reaction mixture was stirred under argon at 85 °C for another 4 hours. Then it was washed with water, the organic layer was dried over Na_2_SO_4_, filtered and the solvent was evaporated under reduced pressure. The residue was purified by column chromatography (silica gel) with CH_2_Cl_2_/petroleum ether (v/v = 1:1) as eluent to provide **4b** (848 mg, 45.7%). ^1^H NMR (600 MHz, CDCl_3_): δ = 7.51-7.47 (m, 2H), 7.24-7.22 (m, 2H), 6.77-6.76 (m, 2H), 6.55 (d, *J* = 4.2 Hz, 2H) ppm.

**Synthesis of 4c**: To a solution of **1c** (1.0 g, 3.8 mmol) in THF (100 mL) was added dropwise a solution of NBS (1.35 g, 7.6 mmol) in THF (20 mL) at -78 °C. The mixture was stirred at -78 °C for 30 min. 2,3-dichloro-5,6-dicyanobenzoquinone (DDQ, 0.95 g, 4.2 mmol) was added and stirring was continued for another 1 hour at room temperature. When the reaction is complete, the excess DDQ is removed with alumina. The organic layer was collected and evaporated under vacuum. The residue was dissolved in 1, 2-dichloroethane (100 mL) and stirred under argon at 50 °C for 30 min. Then Et_3_N (2.2 mL, 15.2 mmol) and BF_3_∙Et_2_O (2.9 mL, 22.8 mmol) were added dropwise, and the reaction mixture was stirred under argon at 85 °C for another 4 hours. Then it was washed with water, the organic layer was dried over Na_2_SO_4_, filtered and the solvent was evaporated under reduced pressure. The residue was purified by column chromatography (silica gel) with CH_2_Cl_2_/petroleum ether (v/v = 1:1) as eluent to provide **4c** (904 mg, 46.2%). ^1^H NMR (600 MHz, CDCl_3_): δ = 6.94 (s, 2H), 6.55 (d, *J* = 4.2 Hz, 2H), 6.46 (d, *J* = 4.2 Hz, 2H), 2.35 (s, 3H), 2.08 (s, 6H) ppm.

**Synthesis of 4d**: To a solution of **1d** (1.0 g, 3.2 mmol) in THF (100 mL) was added dropwise a solution of NBS (1.14 g, 6.4 mmol) in THF (20 mL) at -78 °C. The mixture was stirred at -78 °C for 30 min. 2,3-dichloro-5,6-dicyanobenzoquinone (DDQ, 0.8 g, 3.5 mmol) was added and stirring was continued for another 1 hour at room temperature. When the reaction is complete, the excess DDQ is removed with alumina. The organic layer was collected and evaporated under vacuum. The residue was dissolved in 1, 2-dichloroethane (100 mL) and stirred under argon at 50 °C for 30 min. Then Et_3_N (1.8 mL, 12.8 mmol) and BF_3_∙Et_2_O (2.4 mL, 19.2 mmol) were added dropwise, and the reaction mixture was stirred under argon at 85 °C for another 4 hours. Then it was washed with water, the organic layer was dried over Na_2_SO_4_, filtered and the solvent was evaporated under reduced pressure. The residue was purified by column chromatography (silica gel) with CH_2_Cl_2_/petroleum ether (v/v = 5:2) as eluent to provide **4d** (830 mg, 50.5%). ^1^H NMR (600 MHz, CDCl_3_): δ = 6.66-6.61 (m, 2H), 6.42-6.41 (m, 2H), 6.18 (s, 2H), 3.88 (s, 3H), 3.67 (s, 6H) ppm.

**Synthesis of 5** ^S11^: 1, 8-dibromo-3, 6-di-tert-butyl-9H-carbazole (300 mg, 0.68 mmol), pyrrole-2-boronic acid pinacol ester (400 mg, 2.05 mmol), Pd_2_(dba)_3_ (62 mg, 0.068 mmol), PPh_3_ (71 mg, 0.27 mmol), Cs_2_CO_3_ (442 mg, 1.36 mmol), and CsF (206 mg, 1.36 mmol) were added sequentially to a dry Schlenk reaction tube under nitrogen atmosphere. Toluene (6 mL) and N, N-dimethylformamide (3 mL) were then added under nitrogen protection. The resulting mixture was stirred at 115 °C in an oil bath for 48 h. After cooling, the organic phase was extracted with dichloromethane. The organic phase was then dried with anhydrous sodium sulfate and the solvent removed by rotary evaporation. The residue was purified by column chromatography (silica gel) with CH_2_Cl_2_/petroleum ether (v/v = 2:1) as eluent to provide **5** (98.5 mg, 35.4%). ^1^H NMR (600 MHz, CDCl_3_): δ = 8.67 (s, 1H), 8.58 (s, 2H), 8.03 (d, *J* = 1.8 Hz, 2H), 7.47 (d, *J* = 1.8 Hz, 2H), 6.99-6.98 (m, 2H), 6.68-6.58 (m, 2H), 6.47-6.45 (m, 2H), 1.49 (s, 18H) ppm.

**Synthesis of 6a**: **3a** (218 mg, 0.5 mmol), **5** (204 mg, 0.5 mmol), and Cs_2_CO_3_ (162 mg, 0.5 mmol) were added to a dry Schlenk reaction tube. *p*-xylene (6 mL) under nitrogen protection was then added. The resulting mixture was stirred at 135 °C in an oil bath for 24 h. After cooling, the organic phase was extracted with dichloromethane. The organic phase was then dried with anhydrous sodium sulfate and the solvent removed by rotary evaporation. The residue was purified by column chromatography (silica gel) with CH_2_Cl_2_/petroleum ether (v/v = 1:2) as eluent to provide **6a** (39 mg, 10.2%). ^1^H NMR (600 MHz, CDCl_3_): δ = 11.10 (s, 1H), 9.14 (s, 1H), 8.66 (s, 1H), 8.17 (s, 1H), 8.05 (s, 1H), 7.68 (s, 1H), 7.59 (d, *J* = 21.0 Hz, 2H), 7.34 (d, *J* = 29.4 Hz, 1H), 7.05 (d, *J* = 4.8 Hz, 1H), 6.91 (s, 1H), 6.83-6.81 (m, 2H), 6.67 (s, 1H), 6.49 (s, 1H), 6.43 (s, 1H), 6.38 (s, 1H), 1.52 (d, *J* = 11.4 Hz, 18H) ppm; ^13^C NMR (151 MHz, CDCl_3_): δ = 151.86, 143.85, 143.63, 141.69, 138.81, 136.71, 135.61, 135.57, 132.39, 131.19, 129.82, 125.63, 124.47, 124.37, 124.30, 123.47, 123.36, 123.26, 122.88, 122.44, 122.15, 119.30, 119.19, 117.84, 117.19, 116.30, 114.95, 114.44, 112.15, 110.03, 107.39, 34.80, 31.99, 31.93 ppm; HR-MS (MALDI-TOF-MS) *m/z* = 765.28052 [M]^+^, calcd for (C_43_H_35_BF_7_N_5_)^+^ = 765.2874; UV/Vis (CH_2_Cl_2_): λ_max_ (*ε* [M^-1^cm^-1^]) = 363 (31000), 458 (12000), 647 (70000) nm.

**Synthesis of 6b**: **3b** (182 mg, 0.5 mmol), **5** (204 mg, 0.5 mmol), and Cs_2_CO_3_ (162 mg, 0.5 mmol) were added to a dry Schlenk reaction tube. *p*-xylene (6 mL) under nitrogen protection was then added. The resulting mixture was stirred at 135 °C in an oil bath for 24 h. After cooling, the organic phase was extracted with dichloromethane. The organic phase was then dried with anhydrous sodium sulfate and the solvent removed by rotary evaporation. The residue was purified by column chromatography (silica gel) with CH_2_Cl_2_/petroleum ether (v/v = 1:1) as eluent to provide **6b** (28.8 mg, 8.3%). ^1^H NMR (600 MHz, CDCl_3_): δ = 10.66 (s, 1H), 8.61 (s, 1H), 8.20 (d, *J* = 1.8 Hz, 1H), 8.07 (d, *J* = 1.8 Hz, 1H), 7.89 (d, *J* = 1.8 Hz, 1H), 7.58-7.55 (m, 2H), 7.52 (d, *J* = 1.8 Hz, 1H), 7.23 (d, *J* = 4.8 Hz, 3H), 7.20-7.17 (m, 3H), 6.95 (d, *J* = 4.8 Hz, 1H), 6.89-6.88 (m, 1H), 6.84-6.83 (m, 1H), 6.66-6.65 (m, 1H), 6.38-6.37 (m, 1H), 6.28-6.27 (m, 1H), 6.21-6.20 (m, 1H), 1.52 (s, 9H), 1.51 (s, 9H) ppm; ^13^C NMR (151 MHz, CDCl_3_): δ = 168.21, 164.15, 162.50, 150.06, 143.01, 142.36, 138.16, 136.77, 136.23, 134.95, 133.93, 133.91, 133.17, 133.12, 133.00, 129.77, 128.77, 124.40, 123.78, 123.42, 123.14, 121.24, 120.57, 118.70, 118.67, 117.03, 116.47, 115.04, 114.87, 114.72, 111.36, 110.81, 107.11, 34.80, 32.06, 32.04 ppm; HR-MS (MALDI-TOF-MS) *m/z* = 693.3218 [M]^+^, calcd for (C_43_H_39_BF_3_N_5_)^+^ = 693.3251; UV/Vis (CH_2_Cl_2_): λ_max_ (*ε* [M^-1^cm^-1^]) = 351 (37000), 434 (15000), 629 (69000) nm.

**Synthesis of 6c**: **3c** (194 mg, 0.5 mmol), **5** (204 mg, 0.5 mmol), and Cs_2_CO_3_ (162 mg, 0.5 mmol) were added to a dry Schlenk reaction tube. *p*-xylene (6 mL) under nitrogen protection was then added. The resulting mixture was stirred at 135 °C in an oil bath for 24 h. After cooling, the organic phase was extracted with dichloromethane. The organic phase was then dried with anhydrous sodium sulfate and the solvent removed by rotary evaporation. The residue was purified by column chromatography (silica gel) with CH_2_Cl_2_/petroleum ether (v/v = 1:1) as eluent to provide **6c** (41 mg, 11.5%). ^1^H NMR (600 MHz, CDCl_3_): δ = 11.06-11.04 (m, 1H), 9.24 (s, 1H), 8.63 (s, 1H), 8.14 (s, 1H), 8.05 (s, 1H), 7.68 (s, 1H), 7.58-7.54 (m, 3H), 7.36 (s, 1H), 7.21 (s, 1H), 7.13 (d, *J* = 8.4 Hz, 1H), 6.96 (d, *J* = 27.6 Hz, 3H), 6.85 (s, 1H), 6.80-6.69 (m, 2H), 6.65 (s, 1H), 6.39 (d, *J* = 33.6 Hz, 3H), 2.38 (s, 3H), 2.17 (s, 6H), 1.52 (d, J = 12.6 Hz, 18H) ppm; ^13^C NMR (151 MHz, CDCl_3_): δ = 150.71, 147.62, 147.08, 143.66, 143.54, 139.15, 138.46, 138.41, 138.40, 138.27, 136.87, 136.47, 135.65, 135.55, 133.57, 131.68, 130.35, 130.00, 128.16, 125.40, 124.59, 124.46, 124.43, 124.36, 123.97, 123.56, 123.47, 122.66, 122.07, 121.29, 120.68, 119.08, 117.11, 117.08, 115.97, 115.06, 114.94, 111.06, 109.90, 107.28, 34.85, 34.80, 32.01, 31.96, 31.42, 30.18 ppm; HR-MS (MALDI-TOF-MS) *m/z* = 717.3812 [M]^+^, calcd for (C_46_H_46_BF_2_N_5_)^+^ = 717.3814; UV/Vis (CH_2_Cl_2_): λ_max_ (*ε* [M^-1^cm^-1^]) = 364 (31000), 436 (11000), 623 (72000) nm.

**Synthesis of 6d**: **3d** (218 mg, 0.5 mmol), **5** (204 mg, 0.5 mmol), and Cs_2_CO_3_ (162 mg, 0.5 mmol) were added to a dry Schlenk reaction tube. *p*-xylene (6 mL) under nitrogen protection was then added. The resulting mixture was stirred at 135 °C in an oil bath for 24 h. After cooling, the organic phase was extracted with dichloromethane. The organic phase was then dried with anhydrous sodium sulfate and the solvent removed by rotary evaporation. The residue was purified by column chromatography (silica gel) with CH_2_Cl_2_/petroleum ether (v/v = 3:1) as eluent to provide **6d** (47 mg, 12.3%). ^1^H NMR (600 MHz, CDCl_3_): δ = 11.08-11.04 (m, 1H), 9.29 (s, 1H), 8.63 (s, 1H), 8.12 (d, *J* = 1.8 Hz, 1H), 8.04 (d, *J* = 1.8 Hz, 1H), 7.67 (d, *J* = 1.8 Hz, 1H), 7.60 (d, *J* = 1.8 Hz, 1H), 7.53-7.52 (m, 1H), 7.17-7.16 (m, 1H), 6.92-6.86 (m, 2H), 6.83 (d, *J* = 4.8 Hz, 1H), 6.73-6.72 (m, 1H), 6.67-6.62 (m, 1H), 6.51-6.50 (m, 1H), 6.39-6.30 (m, 2H), 6.24 (s, 2H), 3.91 (s, 3H), 3.72 (s, 6H), 1.53 (s, 9H), 1.51 (s, 9H) ppm; ^13^C NMR (151 MHz, CDCl_3_): δ = 162.35, 159.50, 150.13, 143.57, 143.47, 139.56, 138.56, 135.67, 135.65, 135.51, 134.75, 133.29, 132.31, 130.02, 125.34, 124.69, 124.62, 123.47, 122.69, 122.08, 120.52, 120.00, 119.26, 117.12, 116.87, 115.40, 115.25, 114.88, 110.81, 109.81, 107.26, 104.41, 90.69, 55.93, 55.45, 34.78, 32.01, 31.96 ppm; HR-MS (MALDI-TOF-MS) *m/z* = 765.3669 [M]^+^, calcd for (C_46_H_46_BF_2_N_5_O_3_)^+^ = 765.3662; UV/Vis (CH_2_Cl_2_): λ_max_ (*ε* [M^-1^cm^-1^]) = 364 (28000), 448 (13000), 625 (73000) nm.

**Synthesis of 7a** (**7a** was synthesized according to a method described in ref. S12): **4a** (190 mg, 0.37 mmol), **5** (150 mg, 0.37 mmol), and Cs_2_CO_3_ (240 mg, 0.74 mmol) were added to a dry Schlenk reaction tube. *p*-xylene (6 mL) under nitrogen protection was then added. The resulting mixture was stirred at 135 °C in an oil bath for 48 h. After cooling, the organic phase was extracted with dichloromethane. The organic phase was then dried with anhydrous sodium sulfate and the solvent removed by rotary evaporation. The residue was purified by column chromatography (silica gel) with CH_2_Cl_2_/petroleum ether (v/v = 1:3) as eluent to provide **7a** (89 mg, 31.5%). ^1^H NMR (600 MHz, CD_2_Cl_2_): δ = 9.25 (s, 2H), 8.45 (s, 2H), 8.20 (s, 2H), 7.53-6.91 (m, 8H), 6.85 (s, 1H), 1.62 (s, 18H) ppm; ^13^C NMR (151 MHz, CDCl_3_): δ = 144.45, 143.82, 136.87, 134.86, 134.69, 127.13, 126.00, 124.20, 123.05, 119.14, 117.31, 116.59, 114.48, 113.60, 109.16, 34.90, 32.10 ppm; HR-MS (MALDI-TOF-MS) *m/z* = 763.2717 [M]^+^, calcd for (C_43_H_33_BF_7_N_5_)^+^ = 763.2717; UV/Vis (CH_2_Cl_2_): λ_max_ (*ε* [M^-1^cm^-1^]) = 344 (56000), 575 (29000), 794 (53000) nm.

**Synthesis of 7b**: **4b** (164 mg, 0.37 mmol), **5** (150 mg, 0.37 mmol), and Cs_2_CO_3_ (240 mg, 0.74 mmol) were added to a dry Schlenk reaction tube. *p*-xylene (6 mL) under nitrogen protection was then added. The resulting mixture was stirred at 135 °C in an oil bath for 48 h. After cooling, the organic phase was extracted with dichloromethane. The organic phase was then dried with anhydrous sodium sulfate and the solvent removed by rotary evaporation. The residue was purified by column chromatography (silica gel) with CH_2_Cl_2_/petroleum ether (v/v = 1:2) as eluent to provide **7b** (77 mg, 30.1%). ^1^H NMR (600 MHz, CD_2_Cl_2_): δ = 9.58 (s, 2H), 8.33-8.15 (m, 4H), 7.69 (s, 2H), 7.43-6.87 (m, 11H), 1.59 (s, 18H) ppm; ^13^C NMR (151 MHz, CDCl_3_): δ = 164.34, 162.68, 144.66, 143.87, 134.87, 132.52, 131.15, 128.06, 126.86, 123.15, 118.43, 116.45, 115.57, 115.43, 109.00, 34.90, 32.07 ppm; HR-MS (MALDI-TOF-MS) *m/z* = 691.3097 [M]^+^, calcd for (C_43_H_37_BF_3_N_5_)^+^ = 691.3094; UV/Vis (CH_2_Cl_2_): λ_max_ (*ε* [M^-1^cm^-1^]) = 350 (60000), 553 (34000), 752 (54000) nm.

**Synthesis of 7c**: **4c** (172 mg, 0.37 mmol), **5** (150 mg, 0.37 mmol), and Cs_2_CO_3_ (240 mg, 0.74 mmol) were added to a dry Schlenk reaction tube. *p*-xylene (6 mL) under nitrogen protection was then added. The resulting mixture was stirred at 135 °C in an oil bath for 48 h. After cooling, the organic phase was extracted with dichloromethane. The organic phase was then dried with anhydrous sodium sulfate and the solvent removed by rotary evaporation. The residue was purified by column chromatography (silica gel) with CH_2_Cl_2_/petroleum ether (v/v = 1:2) as eluent to provide **7c** (78 mg, 29.5%). ^1^H NMR (600 MHz, CD_2_Cl_2_): δ = 9.72-9.70 (m, 2H), 8.40 (s, 2H), 8.18 (s, 2H), 7.39 (s, 1H), 7.26-7.14 (m, 6H), 7.06 (s, 2H), 6.82-6.81 (m, 2H), 2.42 (s, 3H), 2.17 (s, 6H), 1.61 (d, *J* = 1.2 Hz, 18H) ppm; ^13^C NMR (151 MHz, CD_2_Cl_2_): δ = 144.75, 144.22, 138.58, 137.39, 134.93, 130.76, 128.25, 127.68, 126.92, 124.92, 123.29, 118.42, 116.75, 116.39, 115.13, 109.22, 34.95, 31.90, 21.03, 19.96 ppm; HR-MS (MALDI-TOF-MS) *m/z* = 715.3687 [M]^+^, calcd for (C_46_H_44_BF_2_N_5_)^+^ = 715.3658; UV/Vis (CH_2_Cl_2_): λ_max_ (*ε* [M^-1^cm^-1^]) = 341 (68000), 547 (35000), 747 (62000) nm.

**Synthesis of 7d**: **4d** (190 mg, 0.37 mmol), **5** (150 mg, 0.37 mmol), and Cs_2_CO_3_ (240 mg, 0.74 mmol) were added to a dry Schlenk reaction tube. *p*-xylene (6 mL) under nitrogen protection was then added. The resulting mixture was stirred at 135 °C in an oil bath for 48 h. After cooling, the organic phase was extracted with dichloromethane. The organic phase was then dried with anhydrous sodium sulfate and the solvent removed by rotary evaporation. The residue was purified by column chromatography (silica gel) with CH_2_Cl_2_/petroleum ether (v/v = 2:1) as eluent to provide **7d** (76 mg, 27.2%). ^1^H NMR (600 MHz, CD_2_Cl_2_): δ = 9.71 (s, 2H), 8.38 (s, 2H), 8.16 (s, 2H), 7.40 (s, 1H), 7.22-7.11 (m, 6H), 6.93 (d, *J* = 4.2 Hz, 2H), 6.34 (s, 2H), 3.94 (s, 3H), 3.75 (s, 6H), 1.60 (s, 18H) ppm; ^13^C NMR (151 MHz, CD_2_Cl_2_): δ = 162.68, 159.85, 144.24, 144.15, 136.64, 135.90, 134.88, 128.79, 128.01, 126.88, 125.02, 123.21, 117.92, 116.63, 116.07, 115.20, 109.09, 104.39, 90.89, 56.08, 55.64, 34.95, 31.91 ppm; HR-MS (MALDI-TOF-MS) *m/z* = 763.3476 [M]^+^, calcd for (C_46_H_44_BF_2_N_5_O_3_)^+^ = 763.3505; UV/Vis (CH_2_Cl_2_): λ_max_ (*ε* [M^-1^cm^-1^]) = 346 (74000), 547 (39000), 748 (62000) nm.

**Heat Stability**

**Figure S1**. TGA graphs of **2a**, **2b**, **2c**, **2d**, **6a**, **6b**, **6c**, **6d**, **7a**, **7b**, **7c** and **7d** with a heating rate of 10 °C min^-1^ under a flow of N_2_.

**NMR Spectra**

**Figure S2**. ^1^H NMR spectrum of **1a** in CDCl_3_.

**Figure S3**. ^1^H NMR spectrum of **1b** in CDCl_3_.

**Figure S4**. ^1^H NMR spectrum of **1c** in CDCl_3_.

**Figure S5**. ^1^H NMR spectrum of **1d** in CDCl_3_.

**Figure S6**. ^1^H NMR spectrum of **2a** in CDCl_3_.

**Figure S7**. ^1^H NMR spectrum of **2b** in CDCl_3_.

**Figure S8**. ^1^H NMR spectrum of **2c** in CDCl_3_.

**Figure S9**. ^1^H NMR spectrum of **2d** in CDCl_3_.

**Figure S10**. ^1^H NMR spectrum of **3a** in CDCl_3_.

**Figure S11**. ^1^H NMR spectrum of **3b** in CDCl_3_.

**Figure S12**. ^1^H NMR spectrum of **3c** in CDCl_3_.

**Figure S13**. ^1^H NMR spectrum of **3d** in CDCl_3_.

**Figure S14**. ^1^H NMR spectrum of **4a** in CDCl_3_.

**Figure S15**. ^1^H NMR spectrum of **4b** in CDCl_3_.

**Figure S16**. ^1^H NMR spectrum of **4c** in CDCl_3_.

**Figure S17**. ^1^H NMR spectrum of **4d** in CDCl_3_.

**Figure S18**. ^1^H NMR spectrum of **5** in CDCl_3_.

**Figure S19**. ^1^H NMR spectrum of **6a** in CDCl_3_.

**Figure S20**. ^1^H NMR spectrum of **6b** in CDCl_3_.

**Figure S21**. ^1^H NMR spectrum of **6c** in CDCl_3_.

**Figure S22**. ^1^H NMR spectrum of **6d** in CDCl_3_.

**Figure S23**. ^1^H NMR spectrum of **7a** in CDCl_3_.

**Figure S24**. ^1^H NMR spectrum of **7b** in CDCl_3_.

**Figure S25**. ^1^H NMR spectrum of **7c** in CDCl_3_.

**Figure S26**. ^1^H NMR spectrum of **7d** in CDCl_3_.

**Figure S27**. ^1^H NMR spectrum of **7a** in CD_2_Cl_2_.

**Figure S28**. ^1^H NMR spectrum of **7b** in CD_2_Cl_2_.

**Figure S29**. ^1^H NMR spectrum of **7c** in CD_2_Cl_2_.

**Figure S30**. ^1^H NMR spectrum of **7d** in CD_2_Cl_2_.

**Figure S31**. ^13^C NMR spectrum of **6a** in CDCl_3_.

**Figure S32**. ^13^C NMR spectrum of **6b** in CDCl_3_.

**Figure S33**. ^13^C NMR spectrum of **6c** in CDCl_3_.

**Figure S34**. ^13^C NMR spectrum of **6d** in CDCl_3_.

**Figure S35**. ^13^C NMR spectrum of **7a** in CDCl_3_.

**Figure S36**. ^13^C NMR spectrum of **7b** in CDCl_3_.

**Figure S37**. ^13^C NMR spectrum of **7c** in CD_2_Cl_2_.

**Figure S38**. ^13^C NMR spectrum of **7d** in CD_2_Cl_2_.

## Mass Spectra

**Figure S39**. HR-MS spectrum of **6a**.

**Figure S40**. HR-MS spectrum of **6b**.

**Figure S41**. HR-MS spectrum of **6c**.

**Figure S42**. HR-MS spectrum of **6d**.

**Figure S43**. HR-MS spectrum of **7a**.

**Figure S44**. HR-MS spectrum of **7b**.

**Figure S45**. HR-MS spectrum of **7c**.

**Figure S46**. HR-MS spectrum of **7d**.

## X-Ray Crystal Date

a)

b)

**Figure S47**. X-ray crystal structure of **7a**. a) Top view, b) side view. The thermal ellipsoids are 50% probability level. H atoms and solvent molecules are omitted for clarity.

**Table S1**. Crystal data and structure refinement for **7a**.

| Identification code | exp_**7a**_sq |
| --- | --- |
| Empirical formula | C_89_H_70_B_2_Cl_3_F_14_N_11_ |
| Formula weight | 1687.53 |
| Temperature/K | 173.00 |
| Crystal system | orthorhombic |
| Space group | Pna2_1_ |
| a/Å | 19.54130(10) |
| b/Å | 14.22100(10) |
| c/Å | 28.40140(10) |
| α/° | 90 |
| β/° | 90 |
| γ/° | 90 |
| Volume/Å^3^ | 7892.66(7) |
| Z | 4 |
| ρ_calc_g/cm^3^ | 1.420 |
| μ/mm^‑1^ | 1.811 |
| F(000) | 3472.0 |
| Crystal size/mm^3^ | 0.4 × 0.3 × 0.2 |
| Radiation | CuKα (λ = 1.54184) |
| 2Θ range for data collection/° | 6.952 to 133.182 |
| Index ranges | -23 ≤ h ≤ 23, -16 ≤ k ≤ 16, -33 ≤ l ≤ 33 |
| Reflections collected | 125544 |
| Independent reflections | 13934 [R_int_ = 0.0501, R_sigma_ = 0.0189] |
| Data/restraints/parameters | 13934/25/1086 |
| Goodness-of-fit on F^2^ | 1.049 |
| Final R indexes [I>=2σ (I)] | R_1_ = 0.0748, wR_2_ = 0.2157 |
| Final R indexes [all data] | R_1_ = 0.0749, wR_2_ = 0.2159 |
| Largest diff. peak/hole / e Å^-3^ | 1.08/-1.01 |
| Flack parameter | 0.49(5) |

CCDC	2405362

## Ground State Structure Optimization

**Figure S48**. Illustration of the optimized ground state structures of molecules **2(a-d)**.

**Figure S49**. Illustration of the optimized ground state structures of molecules **6(a-d)**.

**Figure S50**. Illustration of the optimized ground state structures of molecules **7(a-d)**.

**Table S2**. Theoretical calculation of geometrical parameters (dihedral angles) for BODIPY monomers **2(a-d)**, non-cyclic BODIPY derivatives **6(a-d)** and macrocyclic BODIPY derivatives **7(a-d)**.

|  | Intramolecular dihedral Angle (°) | | | |
| --- | --- | --- | --- | --- |
|  | (ABC)-(R) | (ABC)-(D) | (ABC)-(E) | (ABC)-(FGH) |
| **2a** | 61.26 | / | / | / |
| **2b** | 51.36 | / | / | / |
| **2c** | 89.87 | / | / | / |
| **2d** | 67.96 | / | / | / |
| **6a** | 42.11 | 18.46 | 16.11 | 26.69 |
| **6b** | 34.56 | 20.70 | 16.05 | 26.13 |
| **6c** | 56.50 | 19.21 | 16.96 | 27.24 |
| **6d** | 51.96 | 18.64 | 16.89 | 27.59 |
| **7a** | 61.51 | 28.73 | 28.27 | 16.62 |
| **7b** | 52.95 | 30.29 | 28.12 | 17.37 |
| **7c** | 89.05 | 29.34 | 29.7 | 16.72 |
| **7d** | 70.91 | 30.01 | 29.39 | 17.16 |

## HOMO-LUMO Energy Level Calculation

**Figure S51**. Illustration of the HOMO-LUMO energy level calculation results for molecules **2(a-d)**.

**Figure S52**. Illustration of the HOMO-LUMO energy level calculation results for molecules **6(a-d)**.

**Figure S53**. Illustration of the HOMO-LUMO energy level calculation results for molecules **7(a-d)**.

## Absorption Spectra

**Table S3** Maximum absorption wavelengths of BODIPY derivatives in different solvents

| Compound | Toluene | CHCl_3_ | THF | CHCl_2_ | MeCN |
| --- | --- | --- | --- | --- | --- |
| **6a** | 663 nm | 653 nm | 650 nm | 647 nm | 636 nm |
| **6b** | 648 nm | 639 nm | 638 nm | 629 nm | 625 nm |
| **6c** | 636 nm | 627 nm | 630 nm | 623 nm | 617 nm |
| **6d** | 635 nm | 627 nm | 628 nm | 625 nm | 619 nm |
| **7a** | 799 nm | 796 nm | 795 nm | 794 nm | 785 nm |
| **7b** | 764 nm | 756 nm | 759 nm | 752 nm | 746 nm |
| **7c** | 760 nm | 751 nm | 754 nm | 747 nm | 745 nm |
| **7d** | 757 nm | 749 nm | 749 nm | 748 nm | 745 nm |

**Figure S54**. Long wavelength region of the UV-vis absorption spectra of non-cyclic BODIPY derivatives and cyclic BODIPY derivatives in solvents of varying polarity.

**Fluorescence Spectra**

**Table S4** Optical properties of BODIPY derivatives

| Compounds | *λ_ex_* (nm) | *λ_em_* (nm) | Absolute quantum yield(*Φ*)/% |
| --- | --- | --- | --- |
| **2a** | 500 | 540 | 85.7 |
| **2b** | 500 | 520 | 36.7 |
| **2c** | 500 | 515 | 74.3 |
| **2d** | 500 | 522 | 82.3 |
| **6a** | 630 | / | 0.4 |
| **6b** | 630 | 700 | 2.0 |
| **6c** | 630 | 696 | 4.4 |
| **6d** | 630 | 698 | 6.1 |
| **7a** | 808 | 845 | 4.0 |
| **7b** | 750 | 798 | 11.7 |
| **7c** | 750 | 793 | 12.2 |
| **7d** | 750 | 791 | 13.2 |

The solvent used was dichloromethane, absolute quantum yields measured using an integrating sphere.

**Figure S55**. Fluorescence emission spectra of BODIPY monomers **2** (c = 10^-5^ M; *λ*_ex_ = 500 nm), non-cyclic BODIPY derivatives **6** (c = 10^-5^ M; *λ*_ex_ = 630 nm) and macrocyclic BODIPY derivatives **7** (c = 10^-5^ M; **7a**: *λ*_ex_ = 808 nm; **7b**, **7c**, and **7d**: *λ*_ex_ = 750 nm) in dichloromethane.

## Singlet Oxygen (^1^O_2_) Determination

The ^1^O_2_ generation capacity of **6a**, **6b**, **6c**, **6d**, **7a**, **7b**, **7c** and **7d** were determined with the 9,10-anthracenediylbis(methylene)dimalonic acid (ABDA) as the probe and Methylene Blue (MB) as standard using UV-vis spectroscopy. The UV-vis absorbance intensity of ABDA gradually decreased with the generation of ^1^O_2_. ^1^O_2_ quantum yield was calculated by the following equation:

*Φ*_P_ = *Φ*_MB_k_P_A_MB_/(k_MB_A_P_)

where k_P_ and k_MB_ represent the decomposition rate constants of ABDA with BODIPY derivatives (**6a**, **6b**, **6c**, **6d**, **7a**, **7b**, **7c** and **7d**) and MB (MB), respectively. A_R_ and A_MB_ represent the light absorbed by BODIPY derivatives (**6a**, **6b**, **6c**, **6d**, **7a**, **7b**, **7c** and **7d**) and MB, respectively, which are determined by integration of the areas under the absorption bands in the wavelength range of 400-1000 nm. *Φ*_MB_ is the ^1^O_2_ quantum yield of MB, which is 0.52 in DMSO.^S13^

**Figure S56**. ^1^O_2_ generation of **MB** in DMSO. a) UV-vis spectra of ABDA in the presence of **MB** under Xe lamp (400-1000 nm, 1.0 W/cm^2^) irradiation ([**MB**] = 5 × 10^-6^ M, [ABDA] = 7 × 10^-5^ M); b) Normalized degradation percentages of ABDA at 380 nm under Xe lamp (400-1000 nm, 1.0 W/cm^2^) irradiation ([**MB**] = 5 × 10^-6^ M, [ABDA] = 7 × 10^-5^ M); c) UV-vis spectra of **MB** between 400 and 800 nm ([**MB**] = 5 × 10^-6^ M).

**Figure S57**. ^1^O_2_ generation of non-cyclic BODIPY derivatives (**6a**, **6b**, **6c** and **6d**) in DMSO. a) UV-vis spectra of ABDA in the presence of **6a** under Xe lamp (400-1000 nm, 1.0 W/cm^2^) irradiation ([**6a**] = 5 × 10^-6^ M, [ABDA] = 7 × 10^-5^ M); b) Normalized degradation percentages of ABDA at 380 nm under Xe lamp (400-1000 nm, 1.0 W/cm^2^) irradiation ([**6a**] = 5 × 10^-6^ M, [ABDA] = 7 × 10^-5^ M); c) UV-vis spectra of **6a** between 400 and 800 nm ([**6a**] = 5 × 10^-6^ M); d) UV-vis spectra of ABDA in the presence of **6b** under Xe lamp (400-1000 nm, 1.0 W/cm^2^) irradiation ([**6b**] = 5 × 10^-6^ M, [ABDA] = 7 × 10^-5^ M); e) Normalized degradation percentages of ABDA at 380 nm under Xe lamp (400-1000 nm, 1.0 W/cm^2^) irradiation ([**6b**] = 5 × 10^-6^ M, [ABDA] = 7 × 10^-5^ M); f) UV-vis spectra of **6b** between 400 and 800 nm ([**6b**] = 5 × 10^-6^ M); g) UV-vis spectra of ABDA in the presence of **6c** under Xe lamp (400-1000 nm, 1.0 W/cm^2^) irradiation ([**6c**] = 5 × 10^-6^ M, [ABDA] = 7 × 10^-5^ M); h) Normalized degradation percentages of ABDA at 380 nm under Xe lamp (400-1000 nm, 1.0 W/cm^2^) irradiation ([**6c**] = 5 × 10^-6^ M, [ABDA] = 7 × 10^-5^ M); i) UV-vis spectra of **6c** between 400 and 800 nm ([**6c**] = 5 × 10^-6^ M); j) UV-vis spectra of ABDA in the presence of **6d** under Xe lamp (400-1000 nm, 1.0 W/cm^2^) irradiation ([**6d**] = 5 × 10^-6^ M, [ABDA] = 7 × 10^-5^ M); k) Normalized degradation percentages of ABDA at 380 nm under Xe lamp (400-1000 nm, 1.0 W/cm^2^) irradiation ([**6d**] = 5 × 10^-6^ M, [ABDA] = 7 × 10^-5^ M); l) UV-vis spectra of **6d** between 400 and 800 nm ([**6d**] = 5 × 10^-6^ M).

**Figure S58**. ^1^O_2_ generation of macrocyclic BODIPY derivatives (**7a**, **7b**, **7c** and **7d**) in DMSO. a) UV-vis spectra of ABDA in the presence of **7a** under Xe lamp (400-1000 nm, 1.0 W/cm^2^) irradiation ([**7a**] = 5 × 10^-6^ M, [ABDA] = 7 × 10^-5^ M); b) Normalized degradation percentages of ABDA at 380 nm under Xe lamp (400-1000 nm, 1.0 W/cm^2^) irradiation ([**7a**] = 5 × 10^-6^ M, [ABDA] = 7 × 10^-5^ M); c) UV-vis spectra of **7a** between 400 and 1000 nm ([**7a**] = 5 × 10^-6^ M); d) UV-vis spectra of ABDA in the presence of **7b** under Xe lamp (400-1000 nm, 1.0 W/cm^2^) irradiation ([**7b**] = 5 × 10^-6^ M, [ABDA] = 7 × 10^-5^ M); e) Normalized degradation percentages of ABDA at 380 nm under Xe lamp (400-1000 nm, 1.0 W/cm^2^) irradiation ([**7b**] = 5 × 10^-6^ M, [ABDA] = 7 × 10^-5^ M); f) UV-vis spectra of **7b** between 400 and 1000 nm ([**7b**] = 5 × 10^-6^ M); g) UV-vis spectra of ABDA in the presence of **7c** under Xe lamp (400-1000 nm, 1.0 W/cm^2^) irradiation ([**7c**] = 5 × 10^-6^ M, [ABDA] = 7 × 10^-5^ M); h) Normalized degradation percentages of ABDA at 380 nm under Xe lamp (400-1000 nm, 1.0 W/cm^2^) irradiation ([**7c**] = 5 × 10^-6^ M, [ABDA] = 7 × 10^-5^ M); i) UV-vis spectra of **7c** between 400 and 1000 nm ([**7c**] = 5 × 10^-6^ M); j) UV-vis spectra of ABDA in the presence of **7d** under Xe lamp (400-1000 nm, 1.0 W/cm^2^) irradiation ([**7d**] = 5 × 10^-6^ M, [ABDA] = 7 × 10^-5^ M); k) Normalized degradation percentages of ABDA at 380 nm under Xe lamp (400-1000 nm, 1.0 W/cm^2^) irradiation ([**7d**] = 5 × 10^-6^ M, [ABDA] = 7 × 10^-5^ M); l) UV-vis spectra of **7d** between 400 and 1000 nm ([**7d**] = 5 × 10^-6^ M).

## Synthesis and Characterization of 7a-NPs

**Preparation of 7a-NPs**. **7a** was dissolved in tetrahydrofuran (10 mg, 10 mL) and the solution was then added into PBS standard aqueous solution containing 1,2-distearoyl-sn-glycero-3-phosphoethanolamine-N-[methoxy-(polyethyleneglycol)-2000](DSPE-mPEG_2000_) (50 mg, 10 mL) under sonication. The resulted dispersion was sonicated for 30 min. After that, the mixture was placed on a rotary evaporator for two hours to slowly remove the organic solvents. The remaining 5 mL solution of **7a-NPs** was stored for further tests.

**Figure S59**. Infrared spectra of **7a** (blue) and **7a-NPs** (red).

**Figure S60**. UV-Vis absorption spectra of **7a** in CH_2_Cl_2_ and water (c = 1 × 10^-5^ M).

**Figure S61**. UV-Vis absorption spectra of **7a-NPs** freshly prepared and after 28 days,

Respectively (solvent is aqueous solution of PBS).

## Particle Size Test Method for Nanoparticle 7a-NPs

**Dynamic light scattering：**

The particle size of **7a-NPs** was characterized by a nanoparticle size analyzer (Malvern, Britain). The concentration of **7a-NPs** (2 mg/mL) nanoparticles dispersed in PBS aqueous solution was diluted to 0.01 mg/mL and then tested at 25 °C.

**Transmission electron microscopy：**

Nanoparticle distribution maps of **7a-NPs** were determined by transmission electron microscopy (Tecnai G2 F20 S-TWIN). The sample preparation method was to place the carbon film copper omentum face up on a white filter paper, add 2-3 drops of **7a-NPs** solution (0.01 mg/mL) to the carbon film copper omentum surface, and then test it after natural air drying (4 hours).

## Experimental Data Photothermal Conversion

**Photothermal stability**

Aqueous solution (1.0 mL) of **7a-NPs** with concentration 2 mg/mL was exposed to a laser (808 nm, 2 W cm^-2^, 600 s), and went through three cycles of heating and cooling. The real-time thermal imaging was acquired every 60 sec using a FLIR E5 camera and quantified by FLIR Examiner software. In addition, the photothermal conversion efficiency (η) of **7a-NPs** was calculated according to the literature.^S14,S15^

**Photothermal performance measurement**

Aqueous solutions (1.0 mL) of **7a-NPs** in a quartz cell with different concentrations (0, 30, 90 and 270 μg/mL) were exposed to laser irradiation (808 nm, 2.0 W cm^-2^, 300 s); An aqueous solution (1.0 mL) of **7a-NPs** with a concentration of 270 μg/mL was exposed to a laser of different power (808 nm, 0.5-2 W cm^-2^, 300 s). The temperature was measured every 60 sec. The corresponding infrared thermal images of the sample tubes were also acquired.

**Figure S62.** Thermal images of **7a-NPs** (the concentration is 2 mg/mL) after 808 nm light (2.0 W/cm^2^) irradiation for first heating and cooling.

**Figure S63.** Thermal images of **7a-NPs** (the concentration is 2 mg/mL) after 808 nm light (2.0 W/cm^2^) irradiation for second heating and cooling.

**Figure S64.** Thermal images of **7a-NPs** (the concentration is 2 mg/mL) after 808 nm light (2.0 W/cm^2^) irradiation for third heating and cooling.

**Figure S65.** Thermal images of **7a-NPs** solution (the concentration is 0 μg/mL) after 808 nm light (2.0 W/cm^2^) irradiation.

**Figure S66.** Thermal images of **7a-NPs** solution (the concentration is 30 μg/mL) after 808 nm light (2.0 W/cm^2^) irradiation.

**Figure S67.** Thermal images of **7a-NPs** solution (the concentration is 90 μg/mL) after 808 nm light (2.0 W/cm^2^) irradiation.

**Figure S68.** Thermal images of **7a-NPs** solution (the concentration is 270 μg/mL) after 808 nm light (2.0 W/cm^2^) irradiation.

**Figure S69.** Thermal images of **7a-NPs** solution (the concentration is 270 μg/mL) after 808 nm light (0.5 W/cm^2^) irradiation.

**Figure S70.** Thermal images of **7a-NPs** solution (the concentration is 270 μg/mL) after 808 nm light (1.0 W/cm^2^) irradiation.

**Figure S71.** Thermal images of **7a-NPs** solution (the concentration is 270 μg/mL) after 808 nm light (1.5 W/cm^2^) irradiation.

**Figure S72.** Thermal images of **7a-NPs** solution (the concentration is 270 μg/mL) after 808 nm light (2.0 W/cm^2^) irradiation.

**Figure S73.** a) Temperature profile of **7a-NPs** irradiated by 808 nm laser (2.0 W/cm^2^) for 10 min, followed by natural cooling. b) The linear fitting of time from the cooling period versus negative natural logarithm of driving force temperature for **7a-NPs**.

**Table S5** Summary of photothermal conversion efficiency (PCE) under laser irradiation at 808 nm for recently reported small molecule PTT agents

| Agents | *λ*_Abs_ (nm) | PCE/% | Ref. |
| --- | --- | --- | --- |
| **7a-NPs** | 794 | 44.6 | This work |
| **ICG** | 777 | 15.1 | S16 |
| **NIR6** | 808 | 22.6 | S17 |
| **NIRb6** | 813 | 26.2 | S17 |
| **NIRb10** | 819 | 29.8 | S17 |
| **NIRb14** | 822 | 31.2 | S17 |
| **A1** | 788 | 35 | S18 |
| **BTPETTQ** | 724 | 40 | S19 |
| **4-Pf** | 774 | 41.8 | S20 |
| **2TPE-NDTA** | 700 | 43 | S21 |
| **2TPE-2NDTA** | 700 | 54.9 | S21 |
| **PQDI-NP** | 750 | 64.7 | S22 |
| **IR-Y6** | 730 | 66 | S23 |
| **aza-**  **BODIPY-mPEG** | 842 | 41.4 | S24 |
| **tmf-BDP** | 810 | 88.3 | S25 |
| **QLD-BDP** | 830 | 50.5 | S26 |
| **AzuGIu-BODIPY** | 754 | 51 | S27 |
| **CNTPA** | 929 | 46.2 | S28 |
| **TCF_3_P** | 790 | 94 | S29 |

**Figure S74.** Structures of recently reported small molecule PTT agents.

**Figure S75.** Temperature curve diagram of **7a-NPs** solution (the concentration is 270 μg/mL) and **7a** in DMSO (the concentration is 270 μg/mL) after 808 nm light (2.0 W/cm^2^) irradiation.

**Figure S76.** Thermal images of **7a-NPs** solution (the concentration is 270 μg/mL) after 808 nm light (2.0 W/cm^2^) irradiation.

**Figure S77.** Thermal images of **7a** in DMSO (the concentration is 270 μg/mL) after 808 nm light (2.0 W/cm^2^) irradiation.

**Figure S78.** Temperature rise chart of **7a-NPs** (the concentration is 270 μg/mL) in different dispersion solutions (PBS, Serum (fetal bovine serum), and Medium (Complete culture medium consisting of high glucose medium, fetal bovine serum, L-glutamine, streptomycin sulfate and ampicillin sodium)) after 808 nm light (2.0 W/cm^2^) irradiation (the light duration is 5 min).

**Figure S79.** Thermal images of **7a-NPs** (the concentration is 270 μg/mL) in different dispersion solutions (PBS, Serum, and Medium) after 808 nm light (2.0 W/cm^2^) irradiation (the light duration is 5 min).

## Biological experimental data

**Cell Culture**

A mice breast cancer cell carcinoma (4T1) cell line was cultured in complete PRIM-1640 medium. The medium was supplemented with 10% fetal bovine serum (FBS), 100 units/ml of penicillin, and 100 mg/mL of streptomycin. The cells were maintained in a 37 ^o^C incubator with 5% CO_2_. A human normal breast cell (MCF-10A) line was cultured in complete incubation medium from Shanghai Zhong Qiao Xin Zhou Biotechnology Co., Ltd. The cells were maintained in a 37 ^o^C incubator with 5% CO_2_.

**Cell cytotoxicity assays**

4T1, HUVEC, 3T3 and HL-7702 cells (10000 each) were inoculated in 96-well culture plates at 37 ^o^C and 5% CO_2_ for 24 h. Then the cells were treated with different concentrations of **7a-NPs** (3, 9, 27, 81, and 243 μg/mL). After another 24 h, CCK-8 solution (10 μL) was added, and incubation continued for another 2 h at 37 ^o^C. The optical density was determined at a wavelength of 450 nm using a microplate reader (Multiskan FC, Thermo Fisher, U.S.A.).

**Determination of the antitumor activity in vitro**

The 4T1 cells (100 μL; 8 × 10^4^ cells/mL) were incubated in a 96-well plate for 16 h, enabling them to adhere to the bottom of the plate. Then, the culture medium was replaced with 100 μL of 1640 medium containing PBS and **7a-NPs** (0, 3, 9, 27, 81, and 243 μg/mL) and incubated for another 8 h. After rinsing with PBS, the cells were exposed to light (808 nm, 2.0 W/cm^2^) for 5 min. Then, the optical density was determined at a wavelength of 450 nm using a microplate reader (Multiskan FC, Thermo Fisher, U.S.A.).

**Cell viability detection assay**

Cells were incubated overnight and treated consistently with previous CCK-8 assay. Next, the cells were stained with the Calcein AM/PI. The images were recorded by CLSM.

**In vivo biocompatibility**

All the experimental protocols involving animals were approved by the Medical Ethics Review Committee in Xi’an Medical University (Permit No. XYLS2022188). Female BALB/c nude mice (6-8 weeks old; Xi’an Medical University) were used for the animal studies. **7a-NPs** (Dosage is 25 mg/kg and 50 mg/kg) solution was injected via the tail vein into healthy nude mice; PBS was used as a control. During the experimental period spanning 21 days, mice body weights were measured at days 0, 3, 7, 10, 14, 17, and 21. After 21 days, blood was collected for hematological analysis. The main organs were harvested and stained with Hematoxylin and Eosin (H&E) to co-evaluate the toxicity of **7a-NPs**.

**Tumor mouse culture**

4T1 cells (1 × 10^6^/100 μL) were implanted subcutaneously into the lateral skin of the right hind limb of mice by subcutaneous injection, and intervention began when the average tumor volume reached 40-50 mm^3^.

**Antitumor activity in vivo**

To further evaluate the in vivo therapeutic efficacy, tumor-bearing mice (tumor size 40 mm^3^) were randomly divided into three groups (“Control” (PBS + Light), “**7a-NPs** (0.8 mg/kg) + Light”, and “**7a-NPs** (4.0 mg/kg) + Light”). All animals in each group were injected with the corresponding **7a-NPs** (100 μL, 0.2 mg/mL or 1.0 mg/mL) or PBS solution on day 0, and irradiated using light (808 nm, 2.0 W/cm^2^) 24 h later. During the experimental period spanning 21 days, tumor volumes and mice body weights were measured at days 0, 3, 7, 10, 14, 17, and 21. On the 21st day of treatment, the mice were killed, and the residual tumors were removed and weighed.

**The distribution of 7a-NPs in mice was qualitatively detected by LC-MS**

(1) Experimental material

**Table S6** Reagent consumables

| Reagent | Specification | Rank | Manufacturer |
| --- | --- | --- | --- |
| Formic acid | 98.0% | Mass spectrum level | Sigma-Aldrich |
| Methanol | 4L | Mass spectrum level | Fisher Scientific |
| Ultrapure water | Purified by Millipore pure water system and the resistivity reached 18.2 MΩ·cm. | | |

**Table S7** Principal instrument

| Instrument | Specification and model | Manufacturer |
| --- | --- | --- |
| High performance liquid chromatograph | Vanquish | Thermo Fisher Scientific |
| Mass spectrometer | QE Plus | Thermo Fisher Scientific |
| Centrifuge | D1008E | SCILOGEX |
| Vortex mixer | SCI-VS | SCILOGEX |
| Analytical balance | AL104 | Mettler Toledo Instruments (Shanghai) Co., LTD |
| Constant temperature incubator | DH3600A | Tianjin Test Instrument Co., LTD |
| Vacuum centrifugal concentrator | 268410 | LABCONCO |

(2) Sample acquisition

**7a-NPs** (200 μL, 2 mg/mL) was injected into mice through the tail vein, cultured for 6h and euthanized by CO_2_ inhalation, followed by rapid sampling (liver, kidney, brain, heart, blood).

(3) Sample extraction
1) 0.1 g of liver, kidney, brain and heart samples were weighed, 1 mL methanol was added, and then ground in a grinder until completely chilled, followed by ultrasound for 30 min, short centrifugation to remove large precipitate, 0.42 filter membrane, centrifugation at 12000 rpm for 20 min. Take the supernatant.

2) Take 100 μL of blood sample and add 0.3 mL acetonitrile; Ultrasonic water bath for 30 min, centrifugation for 10 min, 12000 rpm, 4 degrees, supernatant.

(4) LC-MS detection
1) Liquid chromatographic conditions:
a) Analysis column: ACQUUITY^TM^ Premier HSS T3 1.8 μm VanGuard^TM^ FIT 2.1 x 100 mm Column;
b) Column temperature：45 ℃;
c) Mobile phase A: 0.1% formic acid;
d) Mobile phase B: 100% methanol;
e) Flow rate: 0.3 mL/min;
f) UV detector: 254 nm, 268 nm;
g) Analysis time of each component: 16 min.
2) Mass spectrum condition
a) Primary mass spectrum parameters:

Resolution：70,000;

AGCtarget：1e6;

MaximumIT：50ms;

Scanrange：150to1500m/z.

b) Secondary mass spectrum parameters:

Resolution：17,500;

AGCtarget：1e5;

MaximumIT：50ms;

TopN：10;

NCE/steppedNCE：10, 30, 55.

(5) Database Search

The documents acquired by mass spectrometry are passed through the software MS-DIAL 4.70 (MS-DIAL: data independent MS/MS deAvolution for comprehensive metabolome analysis. (Nature Methods, 12, 523-526, 2015) For pre-processing, Including peak extraction, de-noise, deconvolution, peak alignment, derive CSV format 3D data matrix (original data matrix). The peak information extracted was compared with the database, and the three databases MassBank, Respect and GNPS (a total of 14951 records) were searched. This three-dimensional matrix includes information such as sample information, retention time, mass to nucleus ratio and mass spectral response intensity (peak area).

**Table S8** Database matching parameter

| Peak detection parameters | | Alignment parameters setting | | Identification setting | |
| --- | --- | --- | --- | --- | --- |
| Minimum peak height | 1000 amplitude | Retention time tolerance | 0.05 min | Accurate mass tolerance (MS1) | 0.01Da |
| Mass slice width | 0.1 Da | MS1 tolerance | 0.015 Da | Accurate mass tolerance (MS2) | 0.05Da |
|  |  |  |  | Identification score cut off | 80 |

**The distribution of 7a-NPs in mice was quantitatively detected by LC-MS**

(1) Sample acquisition

Four mice were prepared and injected with equal amounts of **7a-NPs** (200 μL, 2 mg/mL) through the tail vein respectively. One mouse was euthanized by CO_2_ inhalation at 6h, 12h, 24h, and 48h after injection of **7a-NPs**, and samples were taken (blood, tumors, muscles).

(2) Experimental purpose

The concentration of **7a** in serum and tissue samples of mice was quantitatively determined.

(3) Standard operating procedures

Sample analysis was carried out according to the internal standard operating procedures of WuXY-POD-PSM-14110 of Chengdu Lilai Biotechnology Co., LTD., China.

(4) Materials and instruments
1) Analyte standard

Name: **7a**

Molecular weight: 763.2717

2) Blank matrix

Mouse serum, tumor homogenate and muscle tissue homogenate.

3) Reagent

Acetonitrile (MeCN): chromatographically pure, Chengdu Cologne;

Methanol (MeOH): chromatographically pure, Merck;

Formic acid (FA): Analytically pure, Chengdu Colon;

Dimethyl sulfoxide (DMSO): Analytically pure, Chengdu Colon;

Ultrapure water: homemade (Chengdu Lilai Biotechnology Co., LTD.)

4) Instrument

LC-MS/MS instrument

Shimadzu liquid chromatography system: high pressure pump (LC-40AC), on-line degasser (DGU-405), automatic sampler (SIL-40CX3), column temperature chamber (CTO-40C), controller (CBM-40).

MS/MS system: Triple Quad 6500^+^ series mass spectrometer with electrospray ionization source, AB SCIEX, USA.

Data acquisition and processing: Analyst 1.7.3 software (AB SCIEX, USA) and Office 2016 (Excel) software (Microsoft, USA).

Analytical Balance: XSR205DU/ Type A Electronic Analytical balance (METTler Toledo, 1/100,000)

Mixer: VOTEX2 S025 mixer, IKA

Centrifuge: Eppendorf/5910R centrifuge (Eppendorf)

Eppendorf/5424R Centrifuge (Eppendorf)

Ultrasonic cleaner: SB-5200DTD (Ningbo Xinzhi Biotechnology Co., LTD.)

Tissue Grinder: SCIENTZ-48(Ningbo Xinzhi Biotechnology Co., LTD.)

Pipette: Eppendorf pipette Rainin pipette

Ultra-pure water machine: H20-ARST-UP-T type (Sartorius)

(5) Experimental process

The linear range of **7a** in serum samples was 1.000~500.0 ng/mL, and the limit of quantification was 1.000 ng/mL.

The linear range of **7a** in tumor homogenate and muscle tissue homogenate samples was 1.000~500.0 ng/mL, and the limit of quantification was 1.000 ng/mL.

The linear range of **7a** in tumor homogenate samples was 500.0~50000 ng/mL (for high concentration samples), and the limit of quantification was 500.0 ng/mL.

1) Chromatographic/mass spectrometry conditions
a) Chromatographic condition

Chromatographic column: ACQUITY UPLC® Protein BEH C4 1.7μm 2.1mm*50mm;

Column temperature: 40°C;

Injection volume: 0.5 μL;

Automatic injector temperature: 10°C;

Running time: 4.00 min;

Flow rate: 0.65 mL/min;

Mobile phase A: aqueous solution containing 0.05% FA

Mobile phase B: acetonitrile solution containing 0.05% FA;

**Table S9** Gradient elution program

| Time (min) | A% | B% |
| --- | --- | --- |
| 0.00 | 55 | 45 |
| 2.20 | 2 | 98 |
| 3.60 | 2 | 98 |
| 3.65 | 55 | 45 |
| 4.00 | 55 | 45 |

b) Mass spectrum condition

Scanning mode: MRM (Multi-Response Monitoring)

Ion polarity: positive mode ionization mode: electrospray ionization (ESI);

CAD: 10psi	CUR: 40psi

GS1:50psi	GS2:50psi

TEM: 450 °C	IS: 5500v

**Table S10** Ion pair information

| Compound | Parent ion (m/z) | Daughter ion (m/z) | Dwell time (ms) | Retention time (min) |
| --- | --- | --- | --- | --- |
| **7a** | 763.3 | 743.3 | 50 | 1.91 |
| Celecoxib | 382.1 | 362.1 | 20 | 0.60 |
| Glyburide | 494.2 | 169.1 | 20 | 0.55 |

2) Working fluid preparation

a) Using 70%MeCN solution as solvent, **7a** standard curve working liquid with concentration of 0.02000~10.00 μg/mL, quality control working liquid with concentration of 0.06000~8.000 μg/mL and 40.00 μg/mL were prepared respectively.

b) DMSO solution was used as solvent to prepare standard curve working liquid with **7a** concentration of 4.000~1000 μg/mL.

3) Standard curve and quality control sample preparation

a) For serum samples, 2 μL of their respective working fluid was taken from 2)-a) and added to 38 μL blank matrix (1mL blank serum was added to 50 μL 30%Tween80). Standard curve samples with concentrations of 1.000~500.0 ng/mL, quality control samples with concentrations of 3.000~400.0 ng/mL (two copies) and 2000 ng/mL quality control samples (three copies) were prepared.

b) For low-concentration tissue samples, 2μL of the respective working liquid in 2)-a) was added to the 38 μL blank tissue matrix to prepare standard curve samples with concentrations ranging from 1.000~500.0 ng/mL. Quality control samples of 3.000~400.0 ng/mL (two samples) and 2000 ng/mL quality control samples (three samples).

c) For high-concentration kidney tissue samples, 2μL of their respective working fluid in 2)-b) was added to 38 μL blank tissue matrix to prepare standard curve samples with concentrations of 200.0~20000 ng/mL.

d) For high-concentration liver tissue, spleen tissue, lung tissue and tumor tissue samples, 2 μL of their respective working fluid in 2)-b) was added to 38 μL blank tissue matrix to prepare standard curve samples with concentrations ranging from 500.0~50,000 ng/mL.

4) Sample handling procedure
a) Serum sample processing

Add 30%Tween80 of 5% of the sample volume to the sample to be tested, and the vortex is reserved;

Samples to be tested, standard curve samples, quality control samples, single blank samples and double blank samples were taken 20 μL and added into 1.5 mL-tube respectively;

600 μL acetonitrile solution containing celecoxib (50 ng/mL) of 1%FA (600 μL acetonitrile was added to the double blank sample) was added to each hole (except the double blank sample hole), vortex for 30s, and centrifuge at high speed (13000 rpm, 4℃) for 15 min;

150 μL supernatant was taken into the 96-deep hole plate, and then LC-MS/MS analysis was performed.

b) Tissue sample processing

After the tissue samples and blank tissues were thawed and restored to room temperature, 4 mL homogenate was added according to 1 g brain tissue (PBS buffer: MeCN (v:v; 2:1)) the proportion of the homogenate is added to the corresponding volume of homogenate.

Low concentration tissue sample processing:

Samples to be tested, standard curve samples, quality control samples, single blank samples and double blank samples were taken 30 μL and added into 1.5 mL-tube respectively;

900 μL MeCN solution containing celecoxib and glyphenuride (50 ng/mL) of 1%FA (900 μL MeCN in the double blank sample) was added to each well (except the double blank sample), swirled for 30s, and centrifuged at high speed (13000 rpm at 4℃) for 15 min;

150 μL supernatant was taken into the 96-deep hole plate, and then LC-MS/MS analysis was performed.

High concentration tissue sample processing:

Samples to be tested, standard curve samples, quality control samples, single blank samples and double blank samples were taken 30 μL and added into 1.5 mL-tube respectively;

900 μL MeCN solution containing celecoxib and glyphenuride (50 ng/mL) of 1%FA (900 μL MeCN in the double blank sample) was added to each well (except the double blank sample), swirled for 30s, and centrifuged at high speed (13000 rpm at 4℃) for 15 min;

50 μL supernatant was taken into 1.5mL-tube containing 950 μL MeCN, swirled for 30s, and centrifuged at high speed (13000 rpm, 4℃) for 2 min;

The last step mixture of 150 μL (3) was taken into the 96-deep hole plate, and then LC-MS/MS analysis was performed.

(6) Result
1) Sensitivity

**Figure S80.** Chromatogram of lower limit of quantification of **7a** in serum matrix (1 ng/mL).

**Figure S81.** Chromatogram of lower limit of quantification of **7a** in tumor homogenate (1 ng/mL).

**Figure S82.** Chromatogram of lower limit of quantification of **7a** in muscle tissue homogenate (1 ng/mL).

**Figure S83.** Chromatogram of lower limit of quantification of **7a** in tumor homogenate (500 ng/mL).

**Table S11** Linear regression equation and lower limit of quantification

| Matrix type | Linear range (ng/mL) | Regression equation | lower limit of quantification (ng/mL) |
| --- | --- | --- | --- |
| Serum | 1.000-500.0 | Y=0.00401x-0.0000753 | 1.000 |
| Tumor | 1.000-500.0 | Y=0.0104x-0.00319 | 1.000 |
| Muscle | 1.000-500.0 | Y=0.0134x-0.00352 | 1.000 |
| Tumor | 500.0-50000 | Y=0.0168x-0.579 | 500.0 |

**Figure S84.** Standard graph of **7a** in serum matrix (R = 0.9965; 1.000-500.0 ng/mL).

**Figure S85.** Standard graph of **7a** in tumor homogenate (R = 0.9916; 1.000-500.0 ng/mL).

**Figure S86.** Standard graph of **7a** in muscle tissue homogenate (R = 0.9913; 1.000-500.0 ng/mL).

**Figure S87.** Standard graph of **7a** in tumor homogenate (R = 0.9986; 500.0-50000 ng/mL).

**Figure S88.** **7a-NPs**, **7a**, and DMSO (243 μg/mL: the content of DMSO is 3%；81 μg/mL: the content of DMSO is 1%；27 μg/mL: the content of DMSO is 0.3%；9 μg/mL: the content of DMSO is 0.1%；3 μg/mL: the content of DMSO is 0.03%) for cytotoxicity detection of 4T1, HUVEC, 3T3, and HL-7702 cells (n = 3).

**Figure S89.** Schematic diagram of hemolysis test results (n = 3).

**The distribution of 7a in mice was qualitatively detected by Uv-Vis-NIR absorption spectroscopy**

Sample acquisition:

**7a-NPs** (200 μL, 2 mg/mL) was injected into mice through the tail vein, cultured for 6h and euthanized by CO_2_ inhalation, followed by rapid sampling (liver, kidney, brain, heart, blood).

Sample extraction:

Liver, kidney, brain and heart samples of 0.1 g were then obtained and 2 mL dichloromethane was added, and then ground in a grinder until completely crushed, followed by ultrasound treatment for 30 min, centrifugation was then used to remove any large precipitate with a 0.42 mum filter membrane. Then the solution was made up to 2 mL for evaluation.

0.1 g of blood was added 10 mL of dichloromethane and 10 mL of normal saline, followed by ultrasound treatment for 30 minutes. After the ultrasonic treatment, the organic phase was separated and the dichloromethane was removed using a rotary evaporator. Then, the remaining substances were dissolved in 2 mL of dichloromethane for evaluation.

**Figure S90.** a) After administering **7a-NPs** via the tail vein to mice then after 6 hours, the absorption spectra of different tissues (liver, brain, kidney, heart, blood) after extraction with dichloromethane were obtained; b) The absorption spectra of different tissues (liver, brain, kidney, heart, blood) of the untreated mice after extraction with dichloromethane.

**Figure S91.** Body weight changes of healthy mice treated with or without **7a-NPs** (n = 4).

**Figure S92.** Blood biochemical test results of mice with or without **7a-NPs** (n = 3).

**Figure S93.** Histological hematoxylin and eosin (H&E) analysis of the major organs (heart,

liver, spleen, lung, and kidney) tissues collected from mice in the different groups with or without **7a-NPs** (24 h and 72h).

**Figure S94.** a) Temperature rise curve of **7a-NPs** (the concentration is 270 μg/mL) with different thickness (1-3 mm) of fat barrier after 808 nm light (2.0 W/cm^2^) irradiation; b) Temperature rise curve of **7a-NPs** (the concentration is 270 μg/mL) with different thickness (1-3 mm) of muscle barrier after 808 nm light (2.0 W/cm^2^) irradiation; c) Temperature rise curves of different concentrations of **7a-NPs** under the skin of mice after 808 nm light (2.0 W/cm^2^) irradiation; d) Temperature rise curves of different concentrations of **7a-NPs** inside mice muscle after 808 nm light (2.0 W/cm^2^) irradiation.

**Figure S95.** Thermal images of **7a-NPs** (the concentration is 270 μg/mL) under 1 mm thickness fat barrier after 808 nm light (2.0 W/cm^2^) irradiation.

**Figure S96.** Thermal images of **7a-NPs** (the concentration is 270 μg/mL) under 2 mm thickness fat barrier after 808 nm light (2.0 W/cm^2^) irradiation.

**Figure S97.** Thermal images of **7a-NPs** (the concentration is 270 μg/mL) under 3 mm thickness fat barrier after 808 nm light (2.0 W/cm^2^) irradiation.

**Figure S98.** Thermal images of **7a-NPs** (the concentration is 270 μg/mL) under 1 mm thickness muscle barrier after 808 nm light (2.0 W/cm^2^) irradiation.

**Figure S99.** Thermal images of **7a-NPs** (the concentration is 270 μg/mL) under 2 mm thickness muscle barrier after 808 nm light (2.0 W/cm^2^) irradiation.

**Figure S100.** Thermal images of **7a-NPs** (the concentration is 270 μg/mL) under 3 mm thickness muscle barrier after 808 nm light (2.0 W/cm^2^) irradiation.

**Figure S101.** Thermal images of **7a-NPs** (the concentration is 30 μg/mL) under the skin of mice after 808 nm light (2.0 W/cm^2^) irradiation.

**Figure S102.** Thermal images of **7a-NPs** (the concentration is 90 μg/mL) under the skin of mice after 808 nm light (2.0 W/cm^2^) irradiation.

**Figure S103.** Thermal images of **7a-NPs** (the concentration is 270 μg/mL) under the skin of mice after 808 nm light (2.0 W/cm^2^) irradiation.

**Figure S104.** Thermal images of **7a-NPs** (the concentration is 30 μg/mL) inside mice muscle after 808 nm light (2.0 W/cm^2^) irradiation.

**Figure S105.** Thermal images of **7a-NPs** (the concentration is 90 μg/mL) inside mice muscle after 808 nm light (2.0 W/cm^2^) irradiation.

**Figure S106.** Thermal images of **7a-NPs** (the concentration is 270 μg/mL) inside mice muscle after 808 nm light (2.0 W/cm^2^) irradiation.

**Figure S107.** a) Schematic diagram illustrating the changes in mice body weight during treatment (n = 6); b) Graph showing the actual weight of the mice (after subtracting the tumor mass) after treatment (n = 6).

**Figure S108.** Schematic diagram of burn section of tumor and surrounding tissue (The dosage of mice was 4.0 mg/kg; The tissue was stained with H&E; The sampling locations of groups **Tumor + Skin** and **Tumor + Muscle** were the central areas of light irradiation, and the sampling locations of groups **Muscle** and **Skin** were the areas around the tumor without light irradiation).

## References

[S1] F. R. Kooriyaden, S. Sujatha, C. Arunkumar, *Polyhedron* **2017**, *128*, 85-94.

[S2] B. Temelli, C. Unaleroglu, *Tetrahedron* **2006**, *62*, 10130-10135.

[S3] Z. Halime, M. Lachkar, N. Matsouki, G. Charalambidis, M. Vaira, A. G. Coutsolelosb, B. Boitrel, *Tetrahedron* **2006**, *62*, 3056-3064.

[S4] X. F. Sheng, W. Wu, Y. S. Qin, X. H. Wang, F. S. Wang,	*Polym. Chem.* **2015**, *6*, 4719-4724.

[S5] A. R. Sekhar, S. K. Sariki, R. V. R. Reddy, A. Bisai, P. K. Sahu, R. S. Tomar, J. Sankar, *Chem. Commun.* **2017**, *53*, 1096-1099.

[S6] X. H. Lv, D. D. Wang, X. H. Chang, H. Y. Cui, CN 119241577 A [P]. 2025-01-03.

[S7] Y. T. Wang, X. G. Shi, C. Y. Yu, Z. K. Zhou, L. Z. Gai, H. Lu, *Dyes Pigments* **2020**, *177*, 108275.

[S8] A. A. Buglak, A. Charisiadis, A. Sheehan, C. J. Kingsbury, M. O. Senge, M. A. Filatov, *Chem. Eur. J.* **2021**, *27*, 9934-9947.

[S9] A. R. Sekhar, M. A. Kaloo, J. Sankar, *Chem. Asian J.* **2014**, *9*, 2422-2426.

[S10] Y. Xu, C. H. Lei, CN 116621866 A [P]. 2023-08-22.

[S11] A. Kalaiselvan, I. S. V. Krishna, A. P. Nambiar, A. Edwin, V. S. Reddy, S. Gokulnath, *Org. Lett.* **2020**, *22*, 4494-4499.

[S12] F. N. Zhang, Y. T. Rao, M. B. Zhou, L. Xu, A. Osuka, J. X. Song, *Chem. Eur. J.* **2023**, *29*, e202302340.

[S13] H. Jung, J. Han, H. Shi, S. Koo, H. Singh, H-J. Kim, J. Sessler, J. Lee, J-H. Kim, J. Kim, *J. Am. Chem. Soc.* **2017**, *139*, 7595−7602.

[S14] Q. Tian, F. Jiang, R. Zou, Q. Liu, Z. Chen, M. Zhu, S. Yang, J. Wang, J. Wang, J. Hu, *ACS Nano* **2011**, *5*, 9761-9771.

[S15] D. Roper, W. Ahn, M. Hoepfner, *J. Phys. Chem. C* **2007**, *111*, 3636-3641.

[S16] M. Zheng, P. Zhao, Z. Luo, P. Gong, C. Zheng, P. Zhang, C. Yue, D. Gao, Y. Ma, L. Cai, *ACS Appl. Mater. Inter.* **2014***, 6,* 6709-6716.

[S17] S. Liu, X. Zhou, H. Zhang, H. Ou, J. Lam, Y. Liu, L. Shi, D. Ding, B. Tang, *J. Am. Chem. Soc.* **2019***, 141,* 5359-5368.

[S18] Y. Xu, T. Feng, T. Yang, H. Wei, H. Yang, G. Li, M. Zhao, S. Liu, W. Huang, Q. Zhao, *ACS Appl. Mater. Inter.* **2018***, 10,* 16299-16307.

[S19] X. Cai, J. Liu, W. Liew, Y. Duan, J. Geng, N. Thakor, K. Yao, L. Liao, B. Liu, *Mater. Chem. Front.* **2017***, 1,* 1556-1562.

[S20] K. Liu, Z. Jiang, R. Lalancette, X. Tang, F. Jäkle, *J. Am. Chem. Soc.* **2022***, 144,* 18908-18917.

[S21] Z. Zhao, C. Chen, W. Wu, F. Wang, L. Du, X. Zhang, Y. Xiong, X. He, Y. Cai, R. Kwok, J. Lam, X. Gao, P. Sun, D. Phillips, D. Ding, B. Tang, *Nat. Commun.* **2019***, 10,* 768.

[S22] C. Liu, S. Zhang, J. Li, J. Wei, K. Müllen, M. Yin, *Angew. Chem. Int. Ed.* **2019***, 58*, 1638-1642.

[S23] H. Gu, W. Liu, S. Zhen, S. Long, W. Sun, J. Cao, X. Zhao, J. Du, J. Fan, X. Peng, *ACS Appl. Mater. Inter.* **2021**, *13,* 46353-46360.

[S24] S. Kampaengsri, K. Chansaenpak, G. Y. Yong, P. Hiranmartsuwan, B. Uengwanarat, R-Y. Lai, P. Meemon, C. S. Kue, A. Kamkaew, *ACS Appl. Bio Mater.* **2022**, *5*, 4567-4577.

[S25] D. M. Xi, M. Xiao, J. F. Cao, L. Y. Zhao, N. Xu, S. R. Long, J. L. Fan, K. Shao, W. Sun, X. H. Yan, X. J. Peng, *Adv. Mater.* **2020**, *32*, 1907855.

[S26] L. L. Cao, Y. K. Li, D. X. Zhang, S. Yue, R. Shang, X. D. Jiang, J. J. Du, *Chin. Chem. Lett.* **2024**, *35*, 109735.

[S27] K. Nishimura, M. Kato, T. Fukui, K. Miura, M. Tsuda, S. Okada, T. Fukushima, H. Nakamura, *Mol. Pharm.* **2025**, *22*, 2660-2670.

[S28] D. An, W. N. Jiang, Z. Y. Zhang, S. P. Wang, W. L. Wang, C. Y. Miao, J. J. Shao, X. C. Dong, *Sens. Actuators B Chem.* **2025**, *442*, 138144.

[S29] G. Kim, Y. Y. Luo, M. Shin, J. Bouffard, J. Bae, Y. Kim, *Adv. Healthcare Mater.* **2024**, 13, 2400885.
